# Development of a validation algorithm for 'present on admission' flagging

**DOI:** 10.1186/1472-6947-9-48

**Published:** 2009-12-01

**Authors:** Terri J Jackson, Jude L Michel, Rosemary Roberts, Jennie Shepheard, Diana Cheng, Julie Rust, Catherine Perry

**Affiliations:** 1 Australian Centre for Economic Research on Health (ACERH), School of Medicine, University of Queensland, Brisbane, Australia; 2 Victorian Department of Health; 50 Lonsdale Street, Melbourne, Victoria, Australia; 3 Health Information Management, La Trobe University, Victoria, Australia; 4 National Centre for Classification in Health (NCCH), Faculty of Health Sciences, The University of Sydney, Lidcombe New South Wales, Australia

## Abstract

**Background:**

The use of routine hospital data for understanding patterns of adverse outcomes has been limited in the past by the fact that pre-existing and post-admission conditions have been indistinguishable. The use of a 'Present on Admission' (or POA) indicator to distinguish pre-existing or co-morbid conditions from those arising during the episode of care has been advocated in the US for many years as a tool to support quality assurance activities and improve the accuracy of risk adjustment methodologies. The USA, Australia and Canada now all assign a flag to indicate the timing of onset of diagnoses. For quality improvement purposes, it is the 'not-POA' diagnoses (that is, those acquired in hospital) that are of interest.

**Methods:**

Our objective was to develop an algorithm for assessing the validity of assignment of 'not-POA' flags. We undertook expert review of the International Classification of Diseases, 10th Revision, Australian Modification (ICD-10-AM) to identify conditions that could not be plausibly hospital-acquired. The resulting computer algorithm was tested against all diagnoses flagged as complications in the Victorian (Australia) Admitted Episodes Dataset, 2005/06. Measures reported include rates of appropriate assignment of the new Australian 'Condition Onset' flag by ICD chapter, and patterns of invalid flagging.

**Results:**

Of 18,418 diagnosis codes reviewed, 93.4% (n = 17,195) reflected agreement on status for flagging by at least 2 of 3 reviewers (including 64.4% unanimous agreement; Fleiss' Kappa: 0.61). In tests of the new algorithm, 96.14% of all hospital-acquired diagnosis codes flagged were found to be valid in the Victorian records analysed. A lower proportion of individual codes was judged to be acceptably flagged (76.2%), but this reflected a high proportion of codes used <5 times in the data set (789/1035 invalid codes).

**Conclusion:**

An indicator variable about the timing of occurrence of diagnoses can greatly expand the use of routinely coded data for hospital quality improvement programmes. The data-cleaning instrument developed and tested here can help guide coding practice in those health systems considering this change in hospital coding. The algorithm embodies principles for development of coding standards and coder education that would result in improved data validity for routine use of non-POA information.

## Background

On 1^st ^October 2007 acute care hospitals in the United States commenced reporting of the 'present on admission' (POA) indicator to distinguish secondary conditions that are present on admission from those arising during the hospital episode. From January 2008 the indicator has been included in the processing of Medicare claims [1]., but its use has been advocated for many years to support quality assurance activities and improve risk adjustment methodologies [2,3]. From October 2008 selected conditions coded as not present on admission do not result in higher payments from Medicare [4].

The quality of routinely collected diagnosis data has become increasingly important, as more clinicians, managers and funders call on these data to answer important questions about health services [5]. While logic edits are quite common in this field [6,7], clinical edits (that apply clinical knowledge to remove implausible coding combinations) are less common. The use of routine hospital data for understanding patterns of adverse outcomes has been limited by the fact that pre-existing and post-admission conditions have been indistinguishable [8-10].

Support for the POA indicator has emerged through US studies which have demonstrated that it improves routine data in various ways [11-18].

Since the mid-1980s, the Victorian Department of Human Services (VDHS) has required all routinely abstracted diagnosis data to be assigned one of four 'prefixes': 'P' for primary diagnoses occasioning the admission and treated during the episode, 'A' for associated pre-existing diagnoses which may have had an impact on treatment even though not actively treated, 'M' for neoplasm morphology codes, and 'C' for diagnoses arising and treated during the current episode [19]--that is 'not present on admission'.

For application of the 'C' prefix (indicating a 'complication'), the coder must ascertain that there was no evidence of the condition existing prior to admission; the C-prefix is used only for a diagnosis arising after admission. This distinguishes incident complications (arising during the current episode of in-hospital care) from those treated in a subsequent episode [20].

In 2007, all Australian states and territories agreed to the adoption of a national 'Condition Onset' flag for diagnoses in the National Inpatient Minimum Dataset, commencing collection in July 2008. Guidance on national condition onset flag assignment has been published in the Australian Coding Standards for ICD-10-AM, 6th edition [21] and broadly follows the Victorian guidelines.

The new national indicators reported in the US differ from those reported in Australia with flags for 'Unknown' and 'Clinically undetermined' in addition to those for 'present on admission' and 'not present on admission'[1]. A list of codes is also published in the ICD-9-CM Official Guidelines for Coding and Reporting which are exempt from POA reporting in the US; these codes are predominantly from the 'factors influencing health status' and 'external cause' chapters [22] of the ICD. In comparison, there are no exempt codes in Australia and a default of 'present on admission' is mandated for conditions where onset is unknown [21].

Canada has adopted a set of 10 'Diagnosis Type' flags, with a 'most responsible diagnosis', external cause, obstetric, and transfer-related markers, in addition to those used in Australia [23]. The national standard for their 'Diagnosis Type 2 Post-Admit Comorbidity' flag specifies 6 sets of excluded codes: neoplasms, diabetes, hypertension, HIV, chronic pulmonary disease, and problems related to medical facilities and other care [24]. When these code sets from the Canadian adaptation of ICD-10 [25] are translated into the Australian version of ICD, they total 962 codes that are disallowed by an edit program used by the Canadian Institute for Health Information (CIHI).

In Victoria, routine diagnosis coding has been validated through coding audits since the mid-1990's [26,27], but assignment of the Victorian prefixes has not been studied. A formal audit (re-abstraction) study including the diagnosis-onset flag was conducted in 2008 as part of the VDHS inpatient data audit program, but has not yet been published [28].

In 2004, VDHS undertook a review of the entire ICD-10-AM classification to clarify which codes could plausibly be assigned to each of the three prefixes (morphologies of neoplasm forming its own distinct part of the classification). No use has been made of this editing algorithm, in part because of the lack of validation of the codes selected by the single reviewer [29]. Subsequently, a warning edit including approximately 2,700 diagnosis codes judged as unlikely to have arisen during a hospital admission has been adopted in Victoria, and alerts hospitals when these codes are erroneously flagged as complications [30].

As more jurisdictions adopt 'present on admission' (POA) or similar timing markers for diagnoses, the need for better measures of data quality becomes apparent. Glance et al. [12,13] validated the POA indicator in Californian hospitals for a range of chronic conditions and reported accuracy between 90-100%. A data cleaning algorithm to reject chronic or congenital conditions flagged as present on admission and exclude them from programs monitoring patient safety would reduce the problem of over-counting of events that has previously been noted in automated surveillance systems [31].

Our goal was to review all 18,418 codes in ICD-10-AM, 4th edition [32] to develop a code set (and computerised algorithm) that could be used by hospitals and health authorities to 'clean' flagged diagnosis data, particularly focussing on codes for slow-developing, chronic and congenital conditions that would never be 'hospital acquired'. The algorithm was also developed as an educational tool for coder training and future coding audit.

## Methods

Three health information managers on the team (JM, RR & JR) completed a survey form to independently evaluate the validity of each ICD-10-AM code for flagging as a diagnosis that might arise during a hospital admission. The draft national guidance, since adopted [21], was used to inform judgements about whether the diagnosis was considered suitable for flagging as non-POA or 'hospital-acquired'.

In the absence of a gold standard against which to compare judgments, a criterion of 2/3 consonant answers was considered sufficient to classify the flagging of the diagnosis as valid or invalid. Reviewers were also given categories for 'Warning' (the code might be used in particular circumstances, but frequent usage would warrant a 'warning' to data managers), and 'don't know' (where the reviewer felt their clinical understanding was not sufficient to make a reliable judgement). These two categories were analysed together, as both indicated uncertainty about assigning a clear include/exclude status. Agreement amongst reviewers was assessed using an online tool to calculate Fleiss' Kappa [33].

Clinician review of codes to be used in the parallel development of a grouping system for hospital-acquired diagnoses [34] identified 61 additional warning codes that were incorporated into the exclusion algorithm.

The algorithm (written in SAS™ coding, see Additional files 1 and 2) was then used to estimate the rate of invalid flagging in the 2005/06 Victorian Admitted Episodes Dataset (VAED) of 2,031,666 de-identified patient episodes from Victorian public and private hospitals. Data custodians waived formal ethical review as the study was a methodological one using de-identified patient data.

## Results

Table 1 shows the distribution of codes by levels of agreement amongst reviewers. Of 18,418 diagnosis codes reviewed, 93.4% (n = 17,195) reflected agreement on status for flagging by at least 2 of the 3 reviewers (including 64.4% unanimous agreement). The remaining 6.6% were a mixture of 'include/warning', 'exclude/warning', 'include/exclude' disagreements and 'don't know' responses that were assigned to a 'warning' class. The Fleiss' Kappa score for agreement amongst reviewers was .61, where .70 is conventionally taken to be adequate agreement. A total of 10,567 codes (57.4%) were designated invalid as hospital-acquired conditions (including 0.03% proposed by the clinical panel). The 1,001 codes designated as 'warning' codes (to be monitored to ensure they are used appropriately) have been combined with the 1,223 codes on which agreement could not be reached for the current version.

**Table 1:** Proportion of codes with substantial reviewer agreement

	Yes/Include	No/Exclude	Warn/Include rarely	TOTAL	%
3 Reviewers Agree	3752	8102	6	11860	64.4%
2 Reviewers Agree	1936	2404	995	5335	29.0%
Mixed disagree/don't know	--	--	1223	1223	6.6%
Further exclusions suggested by clinical panel	--	61	-61	0	

***Total***	***5688***	***10567***	***2163***	***18418***	***100.0%***
	***30.9%***	***57.4%***	***11.7%***	***100.0%***	

Table 2 breaks down the codes nominated for exclusion into their chapters in the ICD, reporting the proportion of codes in the chapter recommended for exclusion. All codes in three chapters of the ICD: 2 (*Neoplasms*), 17 (*Congenital anomalies*) and Appendix A (*Morphologies of neoplasms*), were recommended for exclusion. A further six chapters had more than 50% of codes recommended for exclusion: 21 (*Factors influencing health status*) 93.3%, 13 (*Musculoskeletal*) 60.4%, 4 (*Endocrine*) 59.8%, 11 (*Digestive system*) 57.8%, 5 (*Mental and behavioural*) 57.1, and 20 (*External causes of morbidity*) 51.9%.

**Table 2:** ICD-10-AM Chapters with code numbers excluded in algorithm

Chapter	Code group		Algorithm exclusions	N of codes in chapter	% chapter excluded
Chapter 1	AB	Infectious and parasitic disease	269	766	35.1
Chapter 2	C/D	Neoplasms	790	790	100.0
Chapter 3	part D	Anaemia & other blood diseases	54	164	32.9
Chapter 4	E	Endocrine	274	458	59.8
Chapter 5	F	Mental/behavioural	256	448	57.1
Chapter 6	G	Nervous system	114	387	29.5
Chapter 7/8	H	Eye & ear	75	372	20.2
Chapter 9	I	Circulatory system	142	396	35.9
Chapter 10	J	Respiratory system	76	233	32.6
Chapter 11	K	Digestive system	263	455	57.8
Chapter 12	L	Skin	115	348	33.0
Chapter 13	M	Musculoskeletal system	2031	3361	60.4
Chapter 14	N	Genitourinary system	207	435	47.6
Chapter 15	O	Pregnancy & childbirth	139	436	31.9
Chapter 16	P	Perinatal	115	366	31.4
Chapter 17	Q	Congenital abnormalities	887	887	100.0
Chapter 18	R	Symptoms NEC	6	334	1.8
Chapter 19	S/T	Injuries	44	1785	2.5
Chapter 20	U-Y	External causes' of morbidity	1508	2905	51.9
Chapter 21	Z	Factors influencing health status	638	684	93.3
Appendix A		Morphology of neoplasms	2408	2408	100.0

		Total	10411	18418	56.5

Analysis of the sources of greatest uncertainty amongst reviewers is presented in Table 3. The highest number of mixed response codes was found in the *External Cause *and the *Musculoskeletal System *chapters (Chapters 20 and 13). As a proportion of codes in the chapter, those relating to *Anaemia and Other Blood Diseases *(Chapter 3), *Infectious and Parasitic Diseases *(Chapter 1), and *Nervous System *(Chapter 6) resulted in the greatest uncertainty amongst our reviewers.

**Table 3:** Uncertainty* in condition-onset flagging eligibility by ICD-10 Chapter

Chapter	Code group		Frequency of codes with high uncertainty	N of codes in chapter	%
Chapter 1	AB	Infectious and parasitic disease	138	766	18.0
Chapter 2	C/D	Neoplasms	0	790	0.0
Chapter 3	part D	Anaemia & other blood diseases	30	164	18.3
Chapter 4	E	Endocrine	74	458	16.2
Chapter 5	F	Mental/behavioural	43	448	9.6
Chapter 6	G	Nervous system	66	387	17.1
Chapter 7/8	H	Eye & ear	36	372	9.7
Chapter 9	I	Circulatory system	23	396	5.8
Chapter 10	J	Respiratory system	4	233	1.7
Chapter 11	K	Digestive system	23	455	5.1
Chapter 12	L	Skin	58	348	16.7
Chapter 13	M	Musculoskeletal system	264	3361	7.9
Chapter 14	N	Genitourinary system	49	435	11.3
Chapter 15	O	Pregnancy & childbirth	60	436	13.8
Chapter 16	P	Perinatal	0	366	0.0
Chapter 17	Q	Congenital abnormalities	0	887	0.0
Chapter 18	R	Symptoms NEC	0	334	0.0
Chapter 19	ST	Injuries	64	1785	3.6
Chapter 20	U-Y	External causes of morbidity	275	2905	9.5
Chapter 21	Z	Factors influencing health status	16	684	2.3
Appendix A		Morphology of neoplasms	0	2408	0.0

		***Total***	***1223***	***18418***	***6.6***

*'Uncertainty' defined as <2 reviewers making a positive (valid) or negative (invalid) recommendation.

Table 4 displays the effects of our audit algorithm on code flag status and code-use counts. Victorian coders in 2005-2006 used only 4,345 of the possible 18,418 available ICD-10-AM codes for C-flagged fields (23.6%). Of the codes flagged, 68.0% were determined to be valid using the algorithm, an additional 8.2% of flagged codes would have generated a 'warning' (potentially valid), for a total of 76.2% acceptable code choice. Invalid codes were 23.8% of all individual codes flagged, but of these, 42.0% (n = 433 codes) were used only once in the data set, and 76.5% (n = 789 codes) used in fewer than 5 instances.

**Table 4:** Number of invalid condition onset flags assigned in the Victorian Admitted Episodes Dataset, 2005/06

	**N**	**% Total**	**% Flagged**
	
Total available ICD-10-AM codes	18418	100.0%	
Individual codes C-prefixed	4345	23.6%	100.0%
Valid codes	2956	16.0%	68.0%
Warning codes flagged	357	1.9%	8.2%
Invalid codes flagged	1032	5.6%	23.8%
			
Cases of validly flagged codes	362350	93.9%	
Cases of flagged warning codes	8800	2.3%	96.14%
Cases of invalidly flagged codes	14898	3.9%	
Total cases of C-prefixing	386048	100.0%	

When the number of times a code is used is taken into account, the acceptable coding rate is raised to 96.14% (93.9% valid plus 2.3% 'warning'). Invalid prefixing thus affected 3.9% of the 386,048 diagnoses flagged as 'not-POA' in these 2 million records.

Table 5 breaks down problematic flagging by ICD Chapter. The invalid assignment of the C-prefix/flag by Victorian coders was concentrated in chapters relating to the cardiovascular system (28.5%), the endocrine system (16.0%), the genitourinary system (10.4%) and factors influencing health status (12.2%). In the cardiovascular case, nearly three-quarters of the invalid flagging was for a single code: I10 Essential (primary) hypertension.

**Table 5:** Flagging of 'warning' and invalid codes by ICD Chapter in the Victorian Admitted Episodes Dataset, 2005/06

			**Invalid flagging**		**Warning flagging**	
**Chapter**			**N**	**% Invalid**	**N**	**% Warning**
			
Chapter 1	AB	Infectious and parasitic disease	207	1.4%	11	0.1%
Chapter 2	C/D	Neoplasms	258	1.7%	0	0.0%
Chapter 3	part D	Anaemia & other blood diseases	40	0.3%	66	0.8%
Chapter 4	E	Endocrine	2389	16.0%	4854	55.2%
Chapter 5	F	Mental/behavioural	262	1.8%	477	5.4%
Chapter 6	G	Nervous system	199	1.3%	184	2.1%
Chapter 7/8	H	Eye & ear	174	1.2%	64	0.7%
Chapter 9	I	Circulatory system	4242	28.5%	1068	12.1%
Chapter 10	J	Respiratory system	641	4.3%	20	0.2%
Chapter 11	K	Digestive system	1370	9.2%	165	1.9%
Chapter 12	L	Skin	204	1.4%	243	2.8%
Chapter 13	M	Musculoskeletal system	919	6.2%	709	8.1%
Chapter 14	N	Genitourinary system	1542	10.4%	610	6.9%
Chapter 15	O	Pregnancy & childbirth	13	0.1%	5	0.1%
Chapter 16	P	Perinatal	39	0.3%	14	0.2%
Chapter 17	Q	Congenital abnormalities	26	0.2%	0	0.0%
Chapter 18	R	Symptoms NEC	20	0.1%	0	0.0%
Chapter 19	ST	Injuries	2	0.0%	15	0.2%
Chapter 20	U-Y	'External causes' of morbidity	535	3.6%	138	1.6%
Chapter 21	Z	Factors influencing health status	1815	12.2%	157	1.8%
Appendix A		Morphology of neoplasms	1	0.0%	0	0.0%
			14898	100.0%	8800	100.0%

Flagging of codes relating to factors influencing health status (Chapter 21 of the ICD) reflected considerable confusion, with roughly 20% of invalid codes due to 'hospital-acquired' palliative care and another 20% flagged codes for cancelled procedures. The endocrine system chapter was a large contributor to both invalid flagging and the assignment of the C-prefix/flag to 'warning' codes, representing 16% of the invalids, and 55% of the warnings. In the genitourinary system codes, two-thirds of invalidly flagged cases related to codes for chronic renal failure. The particular issue of acute problems in the context of underlying chronic diseases such as diabetes and renal failure are discussed below, and give rise to much of the confusion in the endocrine and genitourinary chapters of the ICD.

## Discussion

There are many conditions that are unlikely to be hospital-acquired, that is, when detected during a hospital episode, they would inevitably have been present on admission. Our panel of health information managers, supplemented by specialist clinician review, identified 10,567 of the codes in the Australian version of the International Classification of Diseases as unlikely to be hospital-acquired.

When the 2,408 Australian codes used to characterise the morphology of various cancer diagnoses are removed, this results in 8159 diagnosis codes classed as invalid in our algorithm, compared with the current Canadian edit that uses only 962 codes. Comparison with US guidelines for the POA flag is more difficult because the US continues to use the previous version of the ICD (ICD-9-CM).

While the algorithm reported here nominates over half the possible diagnoses as invalid for flagging as hospital-acquired, actual Victorian coding reflects a high degree of selectivity in assigning the C-prefix. Acceptable flagging of diagnosis codes was found in 96.14% of uses in a single year's data. These findings give us confidence in taking a conservative approach to rejecting flagged diagnoses, 'conservative' in the sense of preserving information in the data by defaulting to 'warning' codes.

The patterns of use of invalid and warning codes demonstrated that random error (reflected in single-case misuse) was quite small in comparison with more systematic patterns of invalid flagging. This suggests that revised coding standards for a small number of specific diagnosis codes would yield large improvements in the remaining 3.9% of invalid flag use.

The largest number of invalid flag assignments arose from codes which represent two concepts in a single code. Typical of these codes is E10.64 *Type 1 diabetes mellitus with hypoglycaemia*. Victorian prefixing/flagging rules include a hierarchy which mandates that the P (primary) prefix/flag take precedence over a C prefix/flag when a code contains two concepts (one existing on admission, one being a complication). By making these codes invalid for a C flag, there is a risk that poor diabetes management in hospital, for example, will not be detected. Changing the precedence rule, however, risks over-identifying hospital-acquired complications which would also be undesirable. Resolution of ambiguities in these combined codes is a priority for classification development.

The coding convention of adding a second code to fully describe the 'medical statement' allows for the 'complication' to be coded separately and flagged with C; diabetes with acute renal failure is the best example. Diabetes would be coded to E10.29 *Diabetes with other specified renal complications*, and a second code, N17.9 *Acute renal failure *would be added for the renal complication. Knowledge about areas where such uncertainty exists in the classification can make condition-onset flagging more reliable, and form the basis for better training of coders in recording this valuable information.

The intellectual task of determining which codes may be used legitimately with a 'complications' flag may be compared with that of clinical diagnosis: there will be false positive and false negative assignments, as well as accurately positive and negative ones. False negatives are a well-known limitation of the use of routine hospital data for patient safety research [35]. When medical doctors reviewed subsets of included codes for a parallel project, they recommended more exclusions than our HIM reviewers, who had either disagreed or indicated uncertainty. Of our three reviewers, one had a higher propensity to assign an 'uncertain' status to codes than the other two HIM reviewers, resulting in a slightly lower Kappa value (.61) than desirable. Recognising that many of the codes were split 2/1, where the single reviewer had assigned the conservative 'warning' status, the research team decided that 2 negative recommendations was adequate as the basis for the exclusion algorithm.

We envisage the tool reported here to be subject to ongoing refinement. In particular, prior to 1^st ^July 2007, condition onset was difficult for coders to judge for maternity diagnoses. The Victorian Additions to the Australian Coding Standards [36] changed frequently between the years 2000 and 2007. From 2000 to mid-2004 a limited number of obstetric conditions were permitted to be 'C' flagged; however, from 1^st ^July 2004 all codes assigned for an obstetric episode were directed to be flagged as primary diagnoses, implying they were present on admission. This changed again in July 2006 when conditions or injuries arising after the second stage of labour were considered to be 'complication' diagnoses, that is, arising after the 'admission' of the patient and could be flagged as hospital-acquired.

In practice, coders generally assigned a 'P' (primary) flag to all intrapartum events because of the difficulty of discerning causation by stage of labour. From July 2007 additional instructions for the flagging of obstetric episodes were abandoned and currently obstetric episodes are treated the same as all other episodes of care.

Similar anomalies arise when dealing with neonatal diagnoses. Current definitions for the 'admission' of newborns create difficulties in the assignment of a complications flag for events causing injury to newborns *in hospital *but occurring before birth, the point at which they are deemed to be 'admitted'. When data are used for the screening of adverse events, additional analysis will be required to extract 'primary' diagnoses for neonatal episodes.

Many diagnoses develop over extended time periods, but are not 'chronic' in nature, and additional work may be required to identify which of these conditions might plausibly be hospital-acquired, and over what time scale. One trivial example that arose was whether *L600 Ingrowing nail*, could ever be 'hospital-acquired'. It is assigned 'warning' status, on the reasoning that long stay patients without access to podiatry might develop the condition, rather than being admitted with it. Some 'warning' codes might in future be linked with information on length of stay to better judge the validity of their flagging.

Special consideration was also given to infectious diseases that are typically 'community acquired'. Clinical advisors queried the inclusion of water-borne diseases such as A071 *Giardiasis *and A072 *Cryptosporidiosis*. It was agreed that, although uncommon, these infections could be acquired in a hospital with a compromised water supply, and should be available to coders when documentation showed this to be the case.

The presumption is that most psychiatric conditions will have developed over a period of time prior to hospital admission. However, some medications can give rise to psychiatric symptoms, and hospital care is sometimes itself traumatic. In addition, inappropriate management of drug and alcohol dependence could also give rise to hospital-acquired diagnoses in Chapter 5 of the ICD.

## Conclusion

Indicator variables about the timing of occurrence of diagnoses (pre-existing on hospital admission *vs *newly acquired in a hospital episode) are being introduced in health systems around the world. They can greatly expand the use of routine diagnosis coding for hospital and health system quality improvement programmes.

The data cleaning instrument developed and tested here can help guide coding practice in those health systems introducing this change in hospital coding. It will also be a useful tool for researchers using flagged data to reduce random error in flagging and to target systematic error.

Coding in one Australian state with over 20 years' experience using the flag reflects very high use of valid codes as determined using this algorithm. Revised coding standards and additional coder education would improve data validity for routine use of flagged diagnoses in quality assurance efforts.

## Competing interests

The authors declare that they have no competing interests.

## Authors' contributions

TJJ, as guarantor of this manuscript, was involved in all aspects of the conduct of the research. JLM, RR, JS, JR and DC served as expert reviewers for all or parts of the ICD; CP had the original idea for the study and conducted an earlier pilot of the methods. TJJ and JLM conducted the literature search and drafted the paper; all authors reviewed the manuscript and contributed comments and revisions.

## Pre-publication history

The pre-publication history for this paper can be accessed here:

http://www.biomedcentral.com/1472-6947/9/48/prepub

## Supplementary Material

Additional file 1 **Validation algorithm for 'Present on Admission' (POA) flagging written for SAS™ processing**. This SAS™ program evaluates 40 diagnosis fields in routinely coded hospital data to identify those validly flagged as hospital-acquired ('C' in the data used here). Codes refer to the *International Statistical Classification of Diseases and Related Health Problems, Tenth Revision, Australian Modification (ICD-10-AM)*. Fourth ed. Sydney: National Centre for Classification in Health, The University of Sydney; 2004. See Additional file 2 for further information on the programming. Click here for file

Additional file 2 **Instructions for data cleaning algorithm 20090304**. This document includes additional information for use of the data cleaning algorithm. Click here for file
