# Supplementary material for: Development of a validation algorithm for 'present on admission' flagging
Source: BMC Med Inform Decis Mak. 2009 Dec 1;9:48. doi: 10.1186/1472-6947-9-48 (PMC2793244; doi:10.1186/1472-6947-9-48)
Supplement: Additional file 1 — Validation algorithm for 'Present on Admission' (POA) flagging written for SAS™ processing. This SAS™ program evaluates 40 diagnosis fields in routinely coded hospital data to identify those validly flagged as hospital-acquired ('C' in the data used here). Codes refer to the International Statistical Classification of Diseases and Related Health Problems, Tenth Revision, Australian Modification (ICD-10-AM). Fourth ed. Sydney: National Centre for Classification in Health, The University of Sydney; 2004. See Additional file 2 for further information on the programming. [file 1472-6947-9-48-S1.DOC]

Validation Algorithm for 'Present on Admission' Flagging written for SAS™ processing

**data** combine (compress = yes);

set INSERT DATASET NAME, eg: ‘H:\Vic data\vaed_06_07’;

CodingError =**0**;

sepn=**1**;

array tdx(**40**) tdiag1-tdiag40;

array tpf(**40**) tpref1-tpref40;

do i=**1** to **40** while (tdx(i) ne ' ');

if (substr(tdx(i),**1**,**3**) in

('A33' 'A55' 'A57' 'A58' 'A64' 'A65' 'A70' 'A78' 'B07' 'B20' 'B21' 'B22' 'B24' 'B72' 'B73' 'B91' 'B92' 'C01' 'C07' 'C12' 'C19' 'C20' 'C23' 'C33' 'C37' 'C52' 'C55' 'C56' 'C58' 'C61' 'C64' 'C65' 'C66' 'C73' 'C80' 'D24' 'D27' 'D34' 'D45' 'D66' 'D67' 'E02' 'E40' 'E41' 'E42' 'E43' 'E45' 'E65' 'F21' 'F61' 'F69' 'F82' 'F83' 'F88' 'F89' 'G10' 'G20' 'G22' 'G35' 'H71' 'I00' 'I10' 'J42' 'J47' 'J60' 'J61' 'J64' 'J65' 'K36' 'L80' 'L82' 'L83' 'L84' 'L86' 'N26' 'N40' 'N46' 'N47' 'N62' 'N86' 'N96' 'O11' 'O13' 'O16' 'O40' 'O48' 'O60' 'O80' 'O81' 'O82' 'Q02' 'U71' 'U72' 'V98' 'V99' 'W00' 'W11' 'W12' 'W14' 'W15' 'W16' 'W28' 'W29' 'W32' 'W39' 'W58' 'W69' 'W70' 'W85' 'X22' 'X28' 'X29' 'X30' 'X31' 'X33' 'X35' 'X36' 'X37' 'X52' 'X53' 'X54' 'X57' 'X72' 'X75' 'Y96' 'Y98' 'Z21' 'Z33'))

and tpf(i) eq 'C' then

do;

tpf(i)='*';

CodingError = CodingError+**1**; end;

if (substr(tdx(i),**1**,**4**) in

('A150' 'A151' 'A152' 'A153' 'A154' 'A155' 'A156' 'A157' 'A158' 'A159'

'A160' 'A161' 'A162' 'A163' 'A164' 'A165' 'A167' 'A168' 'A169' 'A170'

'A171' 'A178' 'A179' 'A180' 'A181' 'A182' 'A183' 'A184' 'A185' 'A186'

'A187' 'A188' 'A190' 'A191' 'A192' 'A198' 'A199' 'A393' 'A500' 'A501'

'A502' 'A503' 'A504' 'A505' 'A506' 'A507' 'A509' 'A510' 'A511' 'A512'

'A513' 'A514' 'A515' 'A519' 'A520' 'A521' 'A522' 'A523' 'A527' 'A528'

'A529' 'A530' 'A539' 'A540' 'A541' 'A542' 'A543' 'A544' 'A545' 'A546'

'A548' 'A549' 'A560' 'A561' 'A562' 'A563' 'A564' 'A568' 'A590' 'A598'

'A599' 'A600' 'A601' 'A609' 'A630' 'A638' 'A660' 'A661' 'A662' 'A663'

'A664' 'A665' 'A666' 'A667' 'A668' 'A669' 'A670' 'A671' 'A672' 'A673'

'A679' 'A680' 'A681' 'A689' 'A690' 'A691' 'A692' 'A698' 'A699' 'A710'

'A711' 'A719' 'A740' 'A748' 'A749' 'A750' 'A751' 'A752' 'A753' 'A759'

'A770' 'A771' 'A772' 'A773' 'A778' 'A779' 'A790' 'A791' 'A798' 'A799'

'A810' 'A811' 'A812' 'A818' 'A819' 'A820' 'A821' 'A829' 'A830' 'A831'

'A832' 'A833' 'A835' 'A836' 'A840' 'A841' 'A848' 'A849' 'A950' 'A951'

'A959' 'A960' 'A961' 'A962' 'A968' 'A969' 'A980' 'A981' 'A982' 'A983'

'A984' 'A985' 'B020' 'B021' 'B022' 'B023' 'B027' 'B028' 'B029' 'B180'

'B181' 'B182' 'B188' 'B189' 'B230' 'B238' 'B270' 'B271' 'B334' 'B381'

'B391' 'B401' 'B550' 'B551' 'B552' 'B559' 'B560' 'B561' 'B569' 'B570'

'B571' 'B572' 'B573' 'B574' 'B575' 'B602' 'B650' 'B651' 'B652' 'B653'

'B658' 'B659' 'B660' 'B670' 'B671' 'B672' 'B673' 'B674' 'B675' 'B676'

'B677' 'B678' 'B679' 'B680' 'B681' 'B689' 'B690' 'B691' 'B698' 'B699'

'B700' 'B701' 'B710' 'B711' 'B718' 'B719' 'B740' 'B741' 'B742' 'B743'

'B744' 'B748' 'B749' 'B760' 'B761' 'B768' 'B769' 'B780' 'B781' 'B787'

'B789' 'B834' 'B900' 'B901' 'B902' 'B908' 'B909' 'B940' 'B941' 'B942'

'B948' 'B949' 'C000' 'C001' 'C002' 'C003' 'C004' 'C005' 'C006' 'C008'

'C009' 'C020' 'C021' 'C022' 'C023' 'C024' 'C028' 'C029' 'C030' 'C031'

'C039' 'C040' 'C041' 'C048' 'C049' 'C050' 'C051' 'C052' 'C058' 'C059'

'C060' 'C061' 'C062' 'C068' 'C069' 'C080' 'C081' 'C088' 'C089' 'C090'

'C091' 'C098' 'C099' 'C100' 'C101' 'C102' 'C103' 'C104' 'C108' 'C109'

'C110' 'C111' 'C112' 'C113' 'C118' 'C119' 'C130' 'C131' 'C132' 'C138'

'C139' 'C140' 'C142' 'C148' 'C150' 'C151' 'C152' 'C153' 'C154' 'C155'

'C158' 'C159' 'C160' 'C161' 'C162' 'C163' 'C164' 'C165' 'C166' 'C168'

'C169' 'C170' 'C171' 'C172' 'C173' 'C178' 'C179' 'C180' 'C181' 'C182'

'C183' 'C184' 'C185' 'C186' 'C187' 'C188' 'C189' 'C210' 'C211' 'C212'

'C218' 'C220' 'C221' 'C222' 'C223' 'C224' 'C227' 'C229' 'C240' 'C241'

'C248' 'C249' 'C250' 'C251' 'C252' 'C253' 'C254' 'C257' 'C258' 'C259'

'C260' 'C261' 'C268' 'C269' 'C300' 'C301' 'C310' 'C311' 'C312' 'C313'

'C318' 'C319' 'C320' 'C321' 'C322' 'C323' 'C328' 'C329' 'C340' 'C341'

'C342' 'C343' 'C348' 'C349' 'C380' 'C381' 'C382' 'C383' 'C384' 'C388'

'C390' 'C398' 'C399' 'C400' 'C401' 'C402' 'C403' 'C408' 'C409' 'C411'

'C412' 'C413' 'C414' 'C418' 'C419' 'C430' 'C431' 'C432' 'C433' 'C434'

'C435' 'C436' 'C437' 'C438' 'C439' 'C440' 'C441' 'C442' 'C443' 'C444'

'C445' 'C446' 'C447' 'C448' 'C449' 'C450' 'C451' 'C452' 'C457' 'C459'

'C460' 'C461' 'C462' 'C463' 'C467' 'C468' 'C469' 'C470' 'C471' 'C472'

'C473' 'C474' 'C475' 'C476' 'C478' 'C479' 'C480' 'C481' 'C482' 'C488'

'C490' 'C491' 'C492' 'C493' 'C494' 'C495' 'C496' 'C498' 'C499' 'C500'

'C501' 'C502' 'C503' 'C504' 'C505' 'C506' 'C508' 'C509' 'C510' 'C511'

'C512' 'C518' 'C519' 'C530' 'C531' 'C538' 'C539' 'C540' 'C541' 'C542'

'C543' 'C548' 'C549' 'C570' 'C571' 'C572' 'C573' 'C574' 'C577' 'C578'

'C579' 'C600' 'C601' 'C602' 'C608' 'C609' 'C620' 'C621' 'C629' 'C630'

'C631' 'C632' 'C637' 'C638' 'C639' 'C670' 'C671' 'C672' 'C673' 'C674'

'C675' 'C676' 'C677' 'C678' 'C679' 'C680' 'C681' 'C688' 'C689' 'C690'

'C691' 'C692' 'C693' 'C694' 'C695' 'C696' 'C698' 'C699' 'C700' 'C701'

'C709' 'C710' 'C711' 'C712' 'C713' 'C714' 'C715' 'C716' 'C717' 'C718'

'C719' 'C720' 'C721' 'C722' 'C723' 'C724' 'C725' 'C728' 'C729' 'C740'

'C741' 'C749' 'C750' 'C751' 'C752' 'C753' 'C754' 'C755' 'C758' 'C759'

'C760' 'C761' 'C762' 'C763' 'C764' 'C765' 'C767' 'C768' 'C770' 'C771'

'C772' 'C773' 'C774' 'C775' 'C778' 'C779' 'C780' 'C781' 'C782' 'C783'

'C784' 'C785' 'C786' 'C787' 'C788' 'C790' 'C791' 'C792' 'C793' 'C794'

'C795' 'C796' 'C797' 'C810' 'C811' 'C812' 'C813' 'C817' 'C819' 'C820'

'C821' 'C822' 'C827' 'C829' 'C830' 'C831' 'C832' 'C833' 'C834' 'C835'

'C836' 'C837' 'C838' 'C839' 'C840' 'C841' 'C842' 'C843' 'C844' 'C845'

'C850' 'C851' 'C857' 'C859' 'C960' 'C961' 'C962' 'C963' 'C967' 'C969'

'D000' 'D001' 'D002' 'D010' 'D011' 'D012' 'D013' 'D014' 'D015' 'D017'

'D019' 'D020' 'D021' 'D022' 'D023' 'D024' 'D030' 'D031' 'D032' 'D033'

'D034' 'D035' 'D036' 'D037' 'D038' 'D039' 'D040' 'D041' 'D042' 'D043'

'D044' 'D045' 'D046' 'D047' 'D048' 'D049' 'D050' 'D051' 'D057' 'D059'

'D060' 'D061' 'D067' 'D069' 'D070' 'D071' 'D072' 'D073' 'D074' 'D075'

'D076' 'D090' 'D091' 'D092' 'D093' 'D097' 'D099' 'D100' 'D101' 'D102'

'D103' 'D104' 'D105' 'D106' 'D107' 'D109' 'D110' 'D117' 'D119' 'D120'

'D121' 'D122' 'D123' 'D124' 'D125' 'D126' 'D127' 'D128' 'D129' 'D130'

'D131' 'D132' 'D133' 'D134' 'D135' 'D136' 'D137' 'D139' 'D140' 'D141'

'D142' 'D143' 'D144' 'D150' 'D151' 'D152' 'D157' 'D159' 'D160' 'D161'

'D162' 'D163' 'D165' 'D166' 'D167' 'D168' 'D169' 'D170' 'D171' 'D172'

'D173' 'D174' 'D175' 'D176' 'D177' 'D179' 'D181' 'D190' 'D191' 'D197'

'D199' 'D200' 'D201' 'D210' 'D211' 'D212' 'D213' 'D214' 'D215' 'D216'

'D219' 'D220' 'D221' 'D222' 'D223' 'D224' 'D225' 'D226' 'D227' 'D229'

'D230' 'D231' 'D232' 'D233' 'D234' 'D235' 'D236' 'D237' 'D239' 'D250'

'D251' 'D252' 'D259' 'D260' 'D261' 'D267' 'D269' 'D280' 'D281' 'D282'

'D287' 'D289' 'D290' 'D291' 'D292' 'D293' 'D294' 'D297' 'D299' 'D300'

'D301' 'D302' 'D303' 'D304' 'D307' 'D309' 'D310' 'D311' 'D312' 'D313'

'D314' 'D315' 'D316' 'D319' 'D320' 'D321' 'D329' 'D330' 'D331' 'D332'

'D333' 'D334' 'D337' 'D339' 'D350' 'D351' 'D352' 'D353' 'D354' 'D355'

'D356' 'D357' 'D358' 'D359' 'D360' 'D361' 'D367' 'D369' 'D370' 'D371'

'D372' 'D373' 'D374' 'D375' 'D376' 'D377' 'D379' 'D380' 'D381' 'D382'

'D383' 'D384' 'D385' 'D386' 'D390' 'D391' 'D392' 'D397' 'D399' 'D400'

'D401' 'D407' 'D409' 'D410' 'D411' 'D412' 'D413' 'D414' 'D417' 'D419'

'D420' 'D421' 'D429' 'D430' 'D431' 'D432' 'D433' 'D434' 'D437' 'D439'

'D440' 'D441' 'D442' 'D443' 'D444' 'D445' 'D446' 'D447' 'D448' 'D449'

'D460' 'D461' 'D462' 'D463' 'D464' 'D467' 'D469' 'D470' 'D471' 'D472'

'D473' 'D477' 'D479' 'D480' 'D481' 'D482' 'D483' 'D484' 'D485' 'D486'

'D487' 'D489' 'D560' 'D561' 'D562' 'D563' 'D564' 'D568' 'D569' 'D570'

'D571' 'D572' 'D573' 'D578' 'D580' 'D581' 'D582' 'D588' 'D589' 'D600'

'D610' 'D640' 'D644' 'D680' 'D681' 'D682' 'D720' 'D732' 'D740' 'D750'

'D760' 'D761' 'D800' 'D802' 'D805' 'D814' 'D815' 'D816' 'D817' 'D818'

'D819' 'D820' 'D821' 'D822' 'D823' 'D824' 'D828' 'D829' 'D860' 'D861'

'D862' 'D863' 'D868' 'D869' 'E000' 'E001' 'E002' 'E009' 'E010' 'E011'

'E012' 'E018' 'E030' 'E031' 'E035' 'E040' 'E041' 'E042' 'E048' 'E049'

'E050' 'E051' 'E052' 'E053' 'E062' 'E063' 'E065' 'E071' 'E091' 'E098'

'E099' 'E108' 'E109' 'E118' 'E119' 'E138' 'E139' 'E148' 'E149' 'E200'

'E201' 'E210' 'E220' 'E230' 'E240' 'E250' 'E260' 'E271' 'E282' 'E283'

'E290' 'E300' 'E301' 'E308' 'E309' 'E310' 'E320' 'E340' 'E342' 'E343'

'E344' 'E345' 'E660' 'E661' 'E662' 'E668' 'E669' 'E700' 'E703' 'E710'

'E711' 'E712' 'E713' 'E720' 'E722' 'E723' 'E724' 'E725' 'E730' 'E740'

'E741' 'E742' 'E743' 'E744' 'E748' 'E750' 'E751' 'E752' 'E753' 'E754'

'E755' 'E756' 'E760' 'E761' 'E762' 'E763' 'E770' 'E771' 'E778' 'E779'

'E780' 'E781' 'E782' 'E783' 'E784' 'E785' 'E786' 'E788' 'E789' 'E791'

'E798' 'E799' 'E800' 'E803' 'E804' 'E805' 'E806' 'E840' 'E841' 'E848'

'E849' 'E850' 'E851' 'E852' 'E853' 'E854' 'E858' 'E859' 'F000' 'F001'

'F002' 'F009' 'F011' 'F012' 'F013' 'F018' 'F019' 'F020' 'F021' 'F022'

'F023' 'F024' 'F028' 'F100' 'F101' 'F102' 'F105' 'F106' 'F107' 'F110'

'F111' 'F112' 'F115' 'F116' 'F117' 'F120' 'F121' 'F122' 'F125' 'F126'

'F127' 'F130' 'F131' 'F132' 'F135' 'F136' 'F137' 'F140' 'F141' 'F142'

'F145' 'F146' 'F147' 'F150' 'F151' 'F152' 'F155' 'F156' 'F157' 'F160'

'F161' 'F162' 'F165' 'F166' 'F167' 'F170' 'F171' 'F172' 'F175' 'F176'

'F177' 'F180' 'F181' 'F182' 'F185' 'F186' 'F187' 'F190' 'F191' 'F192'

'F195' 'F196' 'F197' 'F200' 'F201' 'F202' 'F203' 'F204' 'F205' 'F206'

'F208' 'F209' 'F317' 'F334' 'F340' 'F341' 'F348' 'F349' 'F401' 'F402'

'F420' 'F421' 'F422' 'F428' 'F429' 'F500' 'F501' 'F502' 'F503' 'F504'

'F505' 'F508' 'F509' 'F520' 'F521' 'F522' 'F523' 'F524' 'F525' 'F526'

'F527' 'F528' 'F529' 'F550' 'F551' 'F552' 'F553' 'F554' 'F555' 'F556'

'F558' 'F559' 'F600' 'F601' 'F602' 'F604' 'F605' 'F606' 'F607' 'F608'

'F609' 'F620' 'F621' 'F628' 'F629' 'F630' 'F631' 'F632' 'F633' 'F638'

'F639' 'F640' 'F641' 'F642' 'F648' 'F649' 'F650' 'F651' 'F652' 'F653'

'F654' 'F655' 'F656' 'F658' 'F659' 'F660' 'F661' 'F662' 'F668' 'F669'

'F680' 'F681' 'F688' 'F700' 'F701' 'F708' 'F709' 'F710' 'F711' 'F718'

'F719' 'F720' 'F721' 'F728' 'F729' 'F730' 'F731' 'F738' 'F739' 'F780'

'F781' 'F788' 'F789' 'F790' 'F791' 'F798' 'F799' 'F800' 'F801' 'F802'

'F809' 'F810' 'F811' 'F812' 'F813' 'F818' 'F819' 'F840' 'F841' 'F842'

'F843' 'F844' 'F845' 'F848' 'F849' 'F900' 'F901' 'F908' 'F909' 'F910'

'F911' 'F912' 'F913' 'F918' 'F919' 'F920' 'F928' 'F929' 'F930' 'F931'

'F932' 'F933' 'F938' 'F939' 'F940' 'F941' 'F942' 'F948' 'F949' 'F950'

'F951' 'F952' 'F958' 'F959' 'F980' 'F981' 'F982' 'F983' 'F984' 'F985'

'F986' 'F988' 'F989' 'G031' 'G032' 'G110' 'G111' 'G112' 'G113' 'G114'

'G118' 'G119' 'G120' 'G121' 'G122' 'G128' 'G129' 'G130' 'G131' 'G132'

'G138' 'G230' 'G231' 'G232' 'G238' 'G239' 'G241' 'G242' 'G243' 'G244'

'G250' 'G300' 'G301' 'G308' 'G309' 'G310' 'G311' 'G312' 'G313' 'G318'

'G319' 'G320' 'G328' 'G360' 'G370' 'G371' 'G372' 'G373' 'G374' 'G375'

'G378' 'G379' 'G460' 'G461' 'G462' 'G474' 'G478' 'G479' 'G500' 'G501'

'G511' 'G512' 'G513' 'G514' 'G518' 'G519' 'G532' 'G533' 'G550' 'G600'

'G601' 'G602' 'G603' 'G608' 'G609' 'G610' 'G621' 'G631' 'G702' 'G710'

'G712' 'G713' 'G718' 'G719' 'G721' 'G723' 'G731' 'G732' 'G803' 'G804'

'G808' 'G809' 'G900' 'G901' 'G903' 'G930' 'G933' 'G941' 'G942' 'H023'

'H026' 'H027' 'H044' 'H051' 'H053' 'H110' 'H111' 'H112' 'H133' 'H170'

'H171' 'H184' 'H185' 'H186' 'H201' 'H212' 'H214' 'H250' 'H251' 'H252'

'H258' 'H259' 'H260' 'H281' 'H310' 'H311' 'H312' 'H351' 'H353' 'H354'

'H355' 'H400' 'H401' 'H402' 'H420' 'H442' 'H443' 'H445' 'H446' 'H447'

'H472' 'H494' 'H520' 'H521' 'H522' 'H523' 'H524' 'H525' 'H526' 'H527'

'H530' 'H604' 'H612' 'H652' 'H653' 'H654' 'H661' 'H662' 'H663' 'H701'

'H731' 'H740' 'H741' 'H744' 'H800' 'H801' 'H802' 'H808' 'H809' 'H810'

'H911' 'H913' 'H930' 'I010' 'I011' 'I012' 'I018' 'I019' 'I020' 'I029'

'I050' 'I051' 'I052' 'I058' 'I059' 'I060' 'I061' 'I062' 'I068' 'I069'

'I070' 'I071' 'I072' 'I078' 'I079' 'I080' 'I081' 'I082' 'I083' 'I088'

'I089' 'I090' 'I091' 'I092' 'I098' 'I099' 'I110' 'I119' 'I120' 'I129'

'I130' 'I131' 'I132' 'I139' 'I150' 'I151' 'I152' 'I158' 'I200' 'I250'

'I252' 'I253' 'I254' 'I255' 'I256' 'I258' 'I259' 'I271' 'I280' 'I310'

'I311' 'I341' 'I342' 'I350' 'I352' 'I360' 'I362' 'I370' 'I372' 'I420'

'I421' 'I422' 'I423' 'I424' 'I425' 'I426' 'I427' 'I428' 'I429' 'I431'

'I432' 'I510' 'I515' 'I672' 'I673' 'I674' 'I675' 'I680' 'I690' 'I691'

'I692' 'I693' 'I694' 'I698' 'I700' 'I701' 'I708' 'I709' 'I730' 'I731'

'I738' 'I739' 'I773' 'I774' 'I775' 'I780' 'I781' 'I830' 'I831' 'I832'

'I839' 'I840' 'I841' 'I842' 'I843' 'I844' 'I845' 'I846' 'I847' 'I848'

'I849' 'I850' 'I859' 'I860' 'I861' 'I862' 'I863' 'I864' 'I868' 'I872'

'I881' 'I980' 'J310' 'J311' 'J312' 'J320' 'J321' 'J322' 'J323' 'J324'

'J328' 'J329' 'J330' 'J331' 'J338' 'J339' 'J342' 'J343' 'J350' 'J351'

'J352' 'J353' 'J358' 'J359' 'J370' 'J371' 'J381' 'J382' 'J410' 'J411'

'J418' 'J430' 'J431' 'J432' 'J438' 'J439' 'J440' 'J441' 'J448' 'J449'

'J450' 'J451' 'J458' 'J459' 'J620' 'J628' 'J630' 'J631' 'J632' 'J633'

'J634' 'J635' 'J638' 'J660' 'J661' 'J662' 'J668' 'J670' 'J671' 'J672'

'J673' 'J674' 'J675' 'J676' 'J677' 'J678' 'J679' 'J701' 'J703' 'J920'

'J929' 'J961' 'K000' 'K001' 'K002' 'K003' 'K004' 'K005' 'K006' 'K007'

'K008' 'K009' 'K010' 'K011' 'K020' 'K021' 'K022' 'K023' 'K024' 'K028'

'K029' 'K030' 'K031' 'K032' 'K033' 'K034' 'K035' 'K036' 'K037' 'K038'

'K039' 'K042' 'K043' 'K045' 'K046' 'K047' 'K048' 'K051' 'K053' 'K054'

'K060' 'K061' 'K070' 'K071' 'K072' 'K073' 'K074' 'K075' 'K076' 'K078'

'K079' 'K080' 'K082' 'K083' 'K090' 'K091' 'K092' 'K098' 'K099' 'K100'

'K101' 'K110' 'K111' 'K115' 'K132' 'K133' 'K134' 'K135' 'K136' 'K141'

'K142' 'K143' 'K144' 'K145' 'K210' 'K219' 'K220' 'K224' 'K225' 'K230'

'K231' 'K254' 'K255' 'K256' 'K257' 'K264' 'K265' 'K266' 'K267' 'K269'

'K274' 'K275' 'K276' 'K277' 'K283' 'K284' 'K285' 'K286' 'K287' 'K292'

'K293' 'K294' 'K295' 'K311' 'K312' 'K313' 'K314' 'K317' 'K380' 'K381'

'K382' 'K410' 'K411' 'K412' 'K413' 'K414' 'K419' 'K420' 'K421' 'K429'

'K430' 'K431' 'K439' 'K440' 'K441' 'K449' 'K450' 'K451' 'K458' 'K460'

'K461' 'K469' 'K500' 'K501' 'K508' 'K509' 'K510' 'K511' 'K512' 'K513'

'K514' 'K515' 'K518' 'K519' 'K551' 'K580' 'K589' 'K592' 'K593' 'K601'

'K620' 'K621' 'K622' 'K623' 'K634' 'K660' 'K700' 'K701' 'K702' 'K703'

'K704' 'K709' 'K710' 'K711' 'K712' 'K713' 'K714' 'K715' 'K716' 'K717'

'K718' 'K719' 'K721' 'K730' 'K731' 'K732' 'K738' 'K739' 'K740' 'K741'

'K742' 'K743' 'K744' 'K745' 'K746' 'K753' 'K754' 'K760' 'K761' 'K762'

'K764' 'K811' 'K821' 'K824' 'K860' 'K861' 'K900' 'K901' 'K931' 'L100'

'L101' 'L102' 'L103' 'L104' 'L120' 'L121' 'L122' 'L123' 'L128' 'L129'

'L131' 'L400' 'L401' 'L402' 'L403' 'L404' 'L405' 'L408' 'L409' 'L410'

'L411' 'L412' 'L413' 'L414' 'L415' 'L418' 'L419' 'L550' 'L551' 'L552'

'L558' 'L559' 'L570' 'L571' 'L572' 'L573' 'L574' 'L575' 'L578' 'L579'

'L581' 'L602' 'L620' 'L660' 'L670' 'L671' 'L678' 'L679' 'L711' 'L730'

'L731' 'L732' 'L810' 'L811' 'L812' 'L813' 'L814' 'L815' 'L816' 'L817'

'L818' 'L819' 'L850' 'L851' 'L852' 'L853' 'L858' 'L859' 'L870' 'L871'

'L872' 'L878' 'L879' 'L900' 'L901' 'L902' 'L903' 'L904' 'L906' 'L908'

'L909' 'L918' 'L919' 'L920' 'L921' 'L922' 'L930' 'L931' 'L932' 'L940'

'L941' 'L942' 'L943' 'L944' 'L945' 'L946' 'L948' 'L949' 'L950' 'L951'

'L958' 'L959' 'L984' 'M072' 'M083' 'M140' 'M141' 'M143' 'M144' 'M145'

'M146' 'M148' 'M150' 'M151' 'M152' 'M153' 'M154' 'M158' 'M159' 'M160'

'M161' 'M162' 'M163' 'M164' 'M165' 'M166' 'M167' 'M169' 'M170' 'M171'

'M172' 'M173' 'M174' 'M175' 'M179' 'M180' 'M181' 'M182' 'M183' 'M184'

'M185' 'M189' 'M201' 'M202' 'M203' 'M204' 'M214' 'M224' 'M247' 'M312'

'M313' 'M314' 'M321' 'M328' 'M329' 'M332' 'M340' 'M341' 'M348' 'M349'

'M350' 'M351' 'M352' 'M355' 'M356' 'M357' 'M433' 'M434' 'M436' 'M500'

'M501' 'M503' 'M510' 'M511' 'M513' 'M653' 'M654' 'M700' 'M712' 'M720'

'M721' 'M722' 'M750' 'M751' 'M752' 'M753' 'M754' 'M755' 'M762' 'M763'

'M770' 'M771' 'M773' 'M774' 'M852' 'M880' 'M889' 'M910' 'M911' 'M912'

'M913' 'M918' 'M919' 'M920' 'M921' 'M922' 'M923' 'M924' 'M925' 'M926'

'M927' 'M928' 'M929' 'M930' 'M931' 'M938' 'M939' 'M940' 'M941' 'M990'

'M991' 'M992' 'M993' 'M994' 'M995' 'M996' 'M997' 'M998' 'M999' 'N020'

'N021' 'N022' 'N023' 'N024' 'N025' 'N026' 'N027' 'N028' 'N029' 'N030'

'N031' 'N032' 'N033' 'N034' 'N035' 'N036' 'N037' 'N038' 'N039' 'N060'

'N061' 'N062' 'N063' 'N064' 'N065' 'N066' 'N067' 'N068' 'N069' 'N070'

'N071' 'N072' 'N073' 'N074' 'N075' 'N076' 'N077' 'N078' 'N079' 'N110'

'N111' 'N118' 'N119' 'N150' 'N180' 'N188' 'N200' 'N201' 'N202' 'N209'

'N210' 'N211' 'N218' 'N219' 'N220' 'N228' 'N250' 'N251' 'N258' 'N259'

'N270' 'N271' 'N279' 'N281' 'N290' 'N301' 'N302' 'N310' 'N311' 'N312'

'N318' 'N319' 'N323' 'N361' 'N362' 'N411' 'N420' 'N421' 'N422' 'N480'

'N484' 'N486' 'N500' 'N600' 'N601' 'N602' 'N603' 'N604' 'N608' 'N609'

'N642' 'N701' 'N711' 'N731' 'N734' 'N736' 'N740' 'N741' 'N742' 'N743'

'N744' 'N750' 'N761' 'N763' 'N800' 'N801' 'N802' 'N803' 'N804' 'N805'

'N806' 'N808' 'N809' 'N810' 'N811' 'N812' 'N813' 'N814' 'N815' 'N816'

'N818' 'N819' 'N830' 'N831' 'N832' 'N833' 'N834' 'N840' 'N841' 'N842'

'N843' 'N848' 'N849' 'N850' 'N851' 'N852' 'N853' 'N854' 'N855' 'N856'

'N870' 'N871' 'N872' 'N879' 'N880' 'N881' 'N882' 'N883' 'N884' 'N890'

'N891' 'N892' 'N893' 'N894' 'N895' 'N896' 'N897' 'N900' 'N901' 'N902'

'N903' 'N904' 'N905' 'N906' 'N907' 'N910' 'N911' 'N912' 'N913' 'N914'

'N915' 'N920' 'N921' 'N922' 'N923' 'N924' 'N925' 'N926' 'N930' 'N941'

'N942' 'N943' 'N948' 'N949' 'N950' 'N951' 'N952' 'N953' 'N958' 'N959'

'N970' 'N971' 'N972' 'N973' 'N974' 'N978' 'N979' 'O000' 'O001' 'O002'

'O008' 'O009' 'O010' 'O011' 'O019' 'O020' 'O021' 'O028' 'O029' 'O090'

'O091' 'O092' 'O093' 'O094' 'O095' 'O099' 'O100' 'O101' 'O102' 'O103'

'O104' 'O109' 'O120' 'O121' 'O122' 'O220' 'O221' 'O224' 'O240' 'O260'

'O261' 'O262' 'O263' 'O280' 'O281' 'O282' 'O283' 'O284' 'O285' 'O288'

'O289' 'O300' 'O301' 'O302' 'O308' 'O309' 'O310' 'O311' 'O312' 'O320'

'O321' 'O322' 'O323' 'O324' 'O325' 'O326' 'O328' 'O329' 'O330' 'O331'

'O332' 'O333' 'O334' 'O335' 'O336' 'O337' 'O338' 'O339' 'O340' 'O341'

'O342' 'O343' 'O344' 'O345' 'O346' 'O347' 'O348' 'O349' 'O350' 'O351'

'O352' 'O353' 'O354' 'O355' 'O356' 'O357' 'O358' 'O359' 'O360' 'O361'

'O362' 'O363' 'O364' 'O365' 'O366' 'O367' 'O368' 'O369' 'O410' 'O430'

'O431' 'O432' 'O438' 'O439' 'O440' 'O441' 'O981' 'O982' 'O983' 'P000'

'P001' 'P002' 'P003' 'P004' 'P005' 'P006' 'P007' 'P008' 'P009' 'P010'

'P011' 'P012' 'P013' 'P014' 'P015' 'P016' 'P017' 'P018' 'P019' 'P020'

'P021' 'P022' 'P023' 'P024' 'P025' 'P026' 'P027' 'P028' 'P029' 'P030'

'P031' 'P035' 'P036' 'P038' 'P039' 'P040' 'P041' 'P042' 'P043' 'P044'

'P045' 'P046' 'P048' 'P049' 'P050' 'P051' 'P052' 'P059' 'P080' 'P081'

'P082' 'P200' 'P230' 'P231' 'P232' 'P233' 'P234' 'P235' 'P236' 'P238'

'P239' 'P350' 'P351' 'P352' 'P353' 'P358' 'P359' 'P370' 'P371' 'P372'

'P373' 'P374' 'P378' 'P379' 'P500' 'P501' 'P502' 'P503' 'P504' 'P505'

'P508' 'P509' 'P550' 'P551' 'P558' 'P559' 'P560' 'P569' 'P570' 'P612'

'P614' 'P700' 'P701' 'P702' 'P832' 'P835' 'P836' 'P940' 'P941' 'P942'

'P960' 'P963' 'P964' 'Q001' 'Q010' 'Q011' 'Q012' 'Q019' 'Q030' 'Q031'

'Q038' 'Q039' 'Q041' 'Q042' 'Q044' 'Q045' 'Q048' 'Q049' 'Q060' 'Q061'

'Q062' 'Q063' 'Q064' 'Q068' 'Q069' 'Q070' 'Q079' 'Q100' 'Q101' 'Q102'

'Q103' 'Q104' 'Q105' 'Q106' 'Q107' 'Q110' 'Q111' 'Q112' 'Q113' 'Q120'

'Q121' 'Q122' 'Q123' 'Q124' 'Q128' 'Q129' 'Q130' 'Q131' 'Q132' 'Q133'

'Q134' 'Q135' 'Q138' 'Q139' 'Q140' 'Q141' 'Q142' 'Q143' 'Q148' 'Q149'

'Q150' 'Q158' 'Q159' 'Q160' 'Q161' 'Q162' 'Q163' 'Q164' 'Q165' 'Q169'

'Q170' 'Q171' 'Q172' 'Q173' 'Q174' 'Q175' 'Q178' 'Q179' 'Q180' 'Q181'

'Q182' 'Q183' 'Q184' 'Q185' 'Q186' 'Q187' 'Q188' 'Q189' 'Q200' 'Q201'

'Q202' 'Q203' 'Q204' 'Q205' 'Q206' 'Q208' 'Q209' 'Q212' 'Q213' 'Q214'

'Q218' 'Q219' 'Q220' 'Q221' 'Q222' 'Q223' 'Q225' 'Q226' 'Q228' 'Q229'

'Q231' 'Q233' 'Q234' 'Q238' 'Q239' 'Q240' 'Q241' 'Q242' 'Q243' 'Q244'

'Q245' 'Q246' 'Q248' 'Q249' 'Q250' 'Q251' 'Q252' 'Q253' 'Q254' 'Q255'

'Q256' 'Q257' 'Q258' 'Q259' 'Q260' 'Q261' 'Q262' 'Q263' 'Q264' 'Q265'

'Q266' 'Q268' 'Q269' 'Q270' 'Q271' 'Q272' 'Q273' 'Q274' 'Q278' 'Q279'

'Q280' 'Q281' 'Q282' 'Q283' 'Q288' 'Q289' 'Q300' 'Q301' 'Q302' 'Q303'

'Q308' 'Q309' 'Q310' 'Q311' 'Q312' 'Q313' 'Q315' 'Q318' 'Q319' 'Q320'

'Q321' 'Q322' 'Q323' 'Q324' 'Q330' 'Q331' 'Q332' 'Q333' 'Q334' 'Q335'

'Q336' 'Q338' 'Q339' 'Q340' 'Q341' 'Q348' 'Q349' 'Q351' 'Q353' 'Q355'

'Q357' 'Q359' 'Q360' 'Q361' 'Q369' 'Q370' 'Q371' 'Q372' 'Q373' 'Q374'

'Q375' 'Q378' 'Q379' 'Q380' 'Q381' 'Q382' 'Q383' 'Q384' 'Q385' 'Q386'

'Q387' 'Q388' 'Q390' 'Q393' 'Q394' 'Q395' 'Q396' 'Q399' 'Q400' 'Q401'

'Q402' 'Q403' 'Q408' 'Q409' 'Q410' 'Q411' 'Q412' 'Q418' 'Q419' 'Q421'

'Q423' 'Q428' 'Q429' 'Q430' 'Q432' 'Q434' 'Q435' 'Q436' 'Q437' 'Q438'

'Q439' 'Q440' 'Q441' 'Q442' 'Q443' 'Q444' 'Q445' 'Q446' 'Q450' 'Q451'

'Q452' 'Q459' 'Q502' 'Q504' 'Q505' 'Q510' 'Q511' 'Q512' 'Q513' 'Q514'

'Q515' 'Q516' 'Q517' 'Q518' 'Q519' 'Q520' 'Q521' 'Q522' 'Q523' 'Q524'

'Q525' 'Q526' 'Q527' 'Q528' 'Q529' 'Q540' 'Q541' 'Q542' 'Q543' 'Q544'

'Q548' 'Q549' 'Q551' 'Q553' 'Q554' 'Q555' 'Q556' 'Q558' 'Q559' 'Q560'

'Q561' 'Q562' 'Q563' 'Q564' 'Q600' 'Q601' 'Q602' 'Q603' 'Q604' 'Q605'

'Q606' 'Q610' 'Q611' 'Q612' 'Q613' 'Q618' 'Q619' 'Q620' 'Q622' 'Q624'

'Q628' 'Q633' 'Q639' 'Q640' 'Q645' 'Q646' 'Q648' 'Q649' 'Q650' 'Q651'

'Q652' 'Q653' 'Q654' 'Q655' 'Q658' 'Q659' 'Q660' 'Q661' 'Q662' 'Q663'

'Q664' 'Q665' 'Q666' 'Q667' 'Q669' 'Q670' 'Q671' 'Q672' 'Q673' 'Q676'

'Q677' 'Q678' 'Q680' 'Q681' 'Q682' 'Q683' 'Q684' 'Q685' 'Q688' 'Q690'

'Q691' 'Q699' 'Q700' 'Q701' 'Q702' 'Q703' 'Q704' 'Q709' 'Q710' 'Q711'

'Q712' 'Q714' 'Q715' 'Q716' 'Q718' 'Q719' 'Q720' 'Q721' 'Q722' 'Q724'

'Q725' 'Q726' 'Q727' 'Q728' 'Q729' 'Q730' 'Q731' 'Q741' 'Q742' 'Q743'

'Q749' 'Q751' 'Q752' 'Q754' 'Q755' 'Q759' 'Q760' 'Q761' 'Q765' 'Q768'

'Q769' 'Q771' 'Q772' 'Q773' 'Q774' 'Q775' 'Q776' 'Q777' 'Q779' 'Q780'

'Q781' 'Q782' 'Q783' 'Q784' 'Q785' 'Q786' 'Q788' 'Q789' 'Q790' 'Q791'

'Q792' 'Q793' 'Q794' 'Q795' 'Q796' 'Q798' 'Q799' 'Q800' 'Q801' 'Q802'

'Q803' 'Q804' 'Q808' 'Q809' 'Q810' 'Q811' 'Q812' 'Q818' 'Q819' 'Q820'

'Q821' 'Q822' 'Q823' 'Q824' 'Q825' 'Q828' 'Q829' 'Q830' 'Q831' 'Q832'

'Q833' 'Q838' 'Q839' 'Q840' 'Q841' 'Q842' 'Q843' 'Q844' 'Q845' 'Q846'

'Q849' 'Q850' 'Q851' 'Q859' 'Q860' 'Q861' 'Q862' 'Q874' 'Q875' 'Q899'

'Q900' 'Q901' 'Q902' 'Q909' 'Q910' 'Q911' 'Q912' 'Q913' 'Q914' 'Q915'

'Q916' 'Q917' 'Q920' 'Q921' 'Q922' 'Q923' 'Q924' 'Q925' 'Q926' 'Q927'

'Q928' 'Q929' 'Q930' 'Q931' 'Q932' 'Q933' 'Q934' 'Q935' 'Q936' 'Q937'

'Q938' 'Q939' 'Q950' 'Q951' 'Q952' 'Q953' 'Q954' 'Q955' 'Q958' 'Q959'

'Q960' 'Q961' 'Q962' 'Q963' 'Q964' 'Q968' 'Q969' 'Q970' 'Q971' 'Q972'

'Q973' 'Q978' 'Q979' 'Q980' 'Q981' 'Q982' 'Q983' 'Q984' 'Q985' 'Q986'

'Q987' 'Q988' 'Q989' 'Q990' 'Q991' 'Q992' 'Q998' 'Q999' 'R521' 'R522'

'R620' 'R628' 'R629' 'R683' 'T330' 'T331' 'T332' 'T333' 'T334' 'T335'

'T336' 'T337' 'T338' 'T339' 'T340' 'T341' 'T342' 'T343' 'T344' 'T345'

'T346' 'T347' 'T348' 'T349' 'T350' 'T351' 'T352' 'T353' 'T354' 'T355'

'T356' 'T357' 'T635' 'T636' 'T670' 'T671' 'T672' 'T673' 'T674' 'T675'

'T676' 'T677' 'T678' 'T679' 'T690' 'T691' 'T750' 'T751' 'U501' 'U502'

'U504' 'U505' 'U508' 'U509' 'U510' 'U511' 'U513' 'U514' 'U518' 'U519'

'U520' 'U521' 'U528' 'U529' 'U530' 'U531' 'U532' 'U533' 'U534' 'U535'

'U536' 'U537' 'U538' 'U539' 'U542' 'U543' 'U544' 'U546' 'U547' 'U548'

'U549' 'U550' 'U551' 'U553' 'U554' 'U555' 'U556' 'U557' 'U558' 'U559'

'U561' 'U562' 'U564' 'U565' 'U568' 'U569' 'U578' 'U579' 'U580' 'U588'

'U589' 'U590' 'U591' 'U592' 'U593' 'U594' 'U598' 'U599' 'U600' 'U601'

'U603' 'U604' 'U605' 'U606' 'U608' 'U609' 'U610' 'U611' 'U612' 'U615'

'U618' 'U619' 'U620' 'U621' 'U623' 'U624' 'U625' 'U628' 'U629' 'U631'

'U632' 'U633' 'U634' 'U635' 'U636' 'U638' 'U639' 'U640' 'U641' 'U642'

'U643' 'U644' 'U645' 'U646' 'U647' 'U648' 'U649' 'U650' 'U651' 'U652'

'U653' 'U658' 'U659' 'U661' 'U662' 'U663' 'U668' 'U669' 'U670' 'U671'

'U672' 'U673' 'U678' 'U679' 'U681' 'U682' 'U683' 'U684' 'U685' 'U686'

'U688' 'U689' 'U690' 'U691' 'U698' 'U699' 'U700' 'U708' 'U731'

'V010' 'V011' 'V019' 'V020' 'V021' 'V029' 'V030' 'V031'

'V039' 'V040' 'V041' 'V049' 'V050' 'V051' 'V059' 'V060' 'V061' 'V069'

'V090' 'V091' 'V092' 'V093' 'V099' 'V100' 'V101' 'V102' 'V103' 'V104'

'V105' 'V109' 'V110' 'V111' 'V112' 'V113' 'V114' 'V115' 'V119' 'V120'

'V121' 'V122' 'V123' 'V124' 'V125' 'V129' 'V130' 'V131' 'V132' 'V133'

'V134' 'V135' 'V139' 'V140' 'V141' 'V142' 'V143' 'V144' 'V145' 'V149'

'V150' 'V151' 'V152' 'V153' 'V154' 'V155' 'V159' 'V160' 'V161' 'V162'

'V163' 'V164' 'V165' 'V169' 'V170' 'V171' 'V172' 'V173' 'V174' 'V175'

'V179' 'V180' 'V181' 'V182' 'V183' 'V184' 'V185' 'V189' 'V190' 'V191'

'V192' 'V193' 'V194' 'V195' 'V196' 'V198' 'V199' 'V290' 'V291' 'V292'

'V293' 'V294' 'V295' 'V296' 'V298' 'V299' 'V300' 'V301' 'V302' 'V303'

'V304' 'V305' 'V306' 'V307' 'V309' 'V310' 'V311' 'V312' 'V313' 'V314'

'V315' 'V316' 'V317' 'V319' 'V320' 'V321' 'V322' 'V323' 'V324' 'V325'

'V326' 'V327' 'V329' 'V330' 'V331' 'V332' 'V333' 'V334' 'V335' 'V336'

'V337' 'V339' 'V340' 'V341' 'V342' 'V343' 'V344' 'V345' 'V346' 'V347'

'V349' 'V350' 'V351' 'V352' 'V353' 'V354' 'V355' 'V356' 'V357' 'V359'

'V360' 'V361' 'V362' 'V363' 'V364' 'V365' 'V366' 'V367' 'V369' 'V370'

'V371' 'V372' 'V373' 'V374' 'V375' 'V376' 'V377' 'V379' 'V380' 'V381'

'V382' 'V383' 'V384' 'V385' 'V386' 'V387' 'V389' 'V390' 'V391' 'V392'

'V393' 'V394' 'V395' 'V396' 'V398' 'V399' 'V500' 'V501' 'V502' 'V503'

'V504' 'V505' 'V506' 'V507' 'V509' 'V510' 'V511' 'V512' 'V513' 'V514'

'V515' 'V516' 'V517' 'V519' 'V520' 'V521' 'V522' 'V523' 'V524' 'V525'

'V526' 'V527' 'V529' 'V530' 'V531' 'V532' 'V533' 'V534' 'V535' 'V536'

'V537' 'V539' 'V540' 'V541' 'V542' 'V543' 'V544' 'V545' 'V546' 'V547'

'V549' 'V550' 'V551' 'V552' 'V553' 'V554' 'V555' 'V556' 'V557' 'V559'

'V560' 'V561' 'V562' 'V563' 'V564' 'V565' 'V566' 'V567' 'V569' 'V570'

'V571' 'V572' 'V573' 'V574' 'V575' 'V576' 'V577' 'V579' 'V580' 'V581'

'V582' 'V583' 'V584' 'V585' 'V586' 'V587' 'V589' 'V590' 'V591' 'V592'

'V593' 'V594' 'V595' 'V596' 'V598' 'V599' 'V600' 'V601' 'V602' 'V603'

'V604' 'V605' 'V606' 'V607' 'V609' 'V610' 'V611' 'V612' 'V613' 'V614'

'V615' 'V616' 'V617' 'V619' 'V620' 'V621' 'V622' 'V623' 'V624' 'V625'

'V626' 'V627' 'V629' 'V630' 'V631' 'V632' 'V633' 'V634' 'V635' 'V636'

'V637' 'V639' 'V640' 'V641' 'V642' 'V643' 'V644' 'V645' 'V646' 'V647'

'V649' 'V650' 'V651' 'V652' 'V653' 'V654' 'V655' 'V656' 'V657' 'V659'

'V660' 'V661' 'V662' 'V663' 'V664' 'V665' 'V666' 'V667' 'V669' 'V670'

'V671' 'V672' 'V673' 'V674' 'V675' 'V676' 'V677' 'V679' 'V680' 'V681'

'V682' 'V683' 'V684' 'V685' 'V686' 'V687' 'V689' 'V690' 'V691' 'V692'

'V693' 'V694' 'V695' 'V696' 'V698' 'V699' 'V700' 'V701' 'V702' 'V703'

'V704' 'V705' 'V706' 'V707' 'V709' 'V710' 'V711' 'V712' 'V713' 'V714'

'V715' 'V716' 'V717' 'V719' 'V720' 'V721' 'V722' 'V723' 'V724' 'V725'

'V726' 'V727' 'V729' 'V730' 'V731' 'V732' 'V733' 'V734' 'V735' 'V736'

'V737' 'V739' 'V740' 'V741' 'V742' 'V743' 'V744' 'V745' 'V746' 'V747'

'V749' 'V750' 'V751' 'V752' 'V753' 'V754' 'V755' 'V756' 'V757' 'V759'

'V760' 'V761' 'V762' 'V763' 'V764' 'V765' 'V766' 'V767' 'V769' 'V770'

'V771' 'V772' 'V773' 'V774' 'V775' 'V776' 'V777' 'V779' 'V780' 'V781'

'V782' 'V783' 'V784' 'V785' 'V786' 'V787' 'V789' 'V790' 'V791' 'V792'

'V793' 'V794' 'V795' 'V796' 'V798' 'V799' 'V801' 'V802' 'V803' 'V804'

'V805' 'V806' 'V807' 'V808' 'V809' 'V810' 'V811' 'V812' 'V813' 'V814'

'V815' 'V816' 'V817' 'V818' 'V819' 'V820' 'V821' 'V822' 'V823' 'V824'

'V825' 'V826' 'V827' 'V828' 'V829' 'V830' 'V831' 'V832' 'V833' 'V834'

'V835' 'V836' 'V837' 'V839' 'V840' 'V841' 'V842' 'V843' 'V844' 'V845'

'V846' 'V847' 'V849' 'V850' 'V851' 'V852' 'V853' 'V854' 'V855' 'V856'

'V857' 'V859' 'V870' 'V871' 'V872' 'V873' 'V874' 'V875' 'V876' 'V877'

'V878' 'V879' 'V880' 'V881' 'V882' 'V883' 'V884' 'V885' 'V886' 'V887'

'V888' 'V889' 'V890' 'V891' 'V892' 'V893' 'V899' 'V900' 'V901' 'V902'

'V903' 'V904' 'V905' 'V906' 'V907' 'V908' 'V909' 'V910' 'V911' 'V912'

'V913' 'V914' 'V915' 'V916' 'V917' 'V918' 'V919' 'V920' 'V921' 'V922'

'V923' 'V924' 'V925' 'V926' 'V927' 'V928' 'V929' 'V930' 'V931' 'V932'

'V933' 'V934' 'V935' 'V936' 'V937' 'V938' 'V939' 'V940' 'V941' 'V942'

'V943' 'V944' 'V945' 'V946' 'V947' 'V948' 'V949' 'V950' 'V951' 'V952'

'V953' 'V954' 'V958' 'V959' 'V960' 'V961' 'V962' 'V968' 'V969' 'V970'

'V971' 'V972' 'V973' 'V978' 'W020' 'W021' 'W022' 'W023' 'W024' 'W025'

'W090' 'W091' 'W092' 'W093' 'W094' 'W095' 'W096' 'W098' 'W099' 'W210'

'W211' 'W212' 'W218' 'W219' 'W300' 'W301' 'W303' 'W308' 'W309' 'W310'

'W311' 'W312' 'W313' 'W314' 'W318' 'W319' 'W341' 'W342' 'W343' 'W344'

'W349' 'W450' 'W550' 'W558' 'W560' 'W568' 'W569' 'W590' 'W591' 'W598'

'X130' 'X201' 'X262' 'X263' 'X268' 'X269' 'X270' 'X278' 'X670' 'X712'

'X741' 'X742' 'X743' 'X744' 'X749' 'X820' 'X821' 'X822' 'X823' 'X824'

'X825' 'X828' 'X829' 'Y212' 'Y320' 'Y321' 'Y322' 'Y323' 'Y324' 'Y325'

'Y328' 'Y329' 'Y351' 'Y352' 'Y353' 'Y354' 'Y355' 'Y356' 'Y357' 'Y360'

'Y361' 'Y362' 'Y363' 'Y365' 'Y366' 'Y367' 'Y368' 'Y369' 'Y890' 'Y891'

'Y899' 'Y927' 'Y929' 'Z000' 'Z001' 'Z002' 'Z003' 'Z004' 'Z005' 'Z006'

'Z008' 'Z010' 'Z011' 'Z012' 'Z013' 'Z014' 'Z015' 'Z016' 'Z017' 'Z018'

'Z019' 'Z020' 'Z021' 'Z022' 'Z023' 'Z024' 'Z025' 'Z026' 'Z027' 'Z028'

'Z029' 'Z080' 'Z081' 'Z082' 'Z087' 'Z088' 'Z089' 'Z090' 'Z091' 'Z092'

'Z093' 'Z094' 'Z097' 'Z098' 'Z099' 'Z100' 'Z101' 'Z102' 'Z103' 'Z108'

'Z110' 'Z111' 'Z112' 'Z113' 'Z114' 'Z115' 'Z116' 'Z118' 'Z119' 'Z120'

'Z121' 'Z122' 'Z123' 'Z124' 'Z125' 'Z126' 'Z128' 'Z129' 'Z130' 'Z131'

'Z132' 'Z133' 'Z134' 'Z135' 'Z136' 'Z137' 'Z139' 'Z200' 'Z201' 'Z202'

'Z203' 'Z204' 'Z205' 'Z206' 'Z207' 'Z208' 'Z209' 'Z220' 'Z221' 'Z222'

'Z223' 'Z224' 'Z226' 'Z228' 'Z229' 'Z230' 'Z231' 'Z232' 'Z233' 'Z234'

'Z235' 'Z236' 'Z237' 'Z238' 'Z240' 'Z241' 'Z242' 'Z243' 'Z244' 'Z245'

'Z246' 'Z250' 'Z251' 'Z258' 'Z260' 'Z268' 'Z269' 'Z270' 'Z271' 'Z272'

'Z273' 'Z274' 'Z278' 'Z279' 'Z280' 'Z281' 'Z282' 'Z288' 'Z289' 'Z300'

'Z301' 'Z302' 'Z303' 'Z304' 'Z305' 'Z308' 'Z309' 'Z310' 'Z311' 'Z312'

'Z313' 'Z314' 'Z315' 'Z316' 'Z318' 'Z319' 'Z320' 'Z321' 'Z340' 'Z348'

'Z349' 'Z350' 'Z351' 'Z352' 'Z353' 'Z354' 'Z356' 'Z357' 'Z358' 'Z359'

'Z360' 'Z361' 'Z362' 'Z363' 'Z364' 'Z365' 'Z368' 'Z369' 'Z370' 'Z371'

'Z372' 'Z373' 'Z374' 'Z375' 'Z376' 'Z377' 'Z379' 'Z380' 'Z381' 'Z383'

'Z384' 'Z386' 'Z387' 'Z391' 'Z392' 'Z408' 'Z409' 'Z410' 'Z411' 'Z412'

'Z413' 'Z418' 'Z419' 'Z420' 'Z421' 'Z422' 'Z423' 'Z424' 'Z428' 'Z429'

'Z430' 'Z431' 'Z432' 'Z433' 'Z434' 'Z435' 'Z436' 'Z437' 'Z438' 'Z439'

'Z440' 'Z441' 'Z442' 'Z443' 'Z448' 'Z449' 'Z450' 'Z451' 'Z452' 'Z453'

'Z458' 'Z459' 'Z460' 'Z461' 'Z462' 'Z463' 'Z464' 'Z465' 'Z466' 'Z467'

'Z468' 'Z469' 'Z470' 'Z478' 'Z479' 'Z480' 'Z488' 'Z489' 'Z490' 'Z491'

'Z492' 'Z500' 'Z501' 'Z502' 'Z503' 'Z504' 'Z505' 'Z506' 'Z507' 'Z508'

'Z509' 'Z510' 'Z511' 'Z513' 'Z514' 'Z515' 'Z519' 'Z521' 'Z522' 'Z523'

'Z524' 'Z525' 'Z526' 'Z527' 'Z528' 'Z529' 'Z530' 'Z531' 'Z532' 'Z538'

'Z539' 'Z540' 'Z541' 'Z542' 'Z543' 'Z544' 'Z547' 'Z548' 'Z549' 'Z550'

'Z551' 'Z552' 'Z553' 'Z554' 'Z558' 'Z559' 'Z560' 'Z561' 'Z562' 'Z563'

'Z564' 'Z565' 'Z566' 'Z567' 'Z570' 'Z571' 'Z572' 'Z573' 'Z574' 'Z575'

'Z576' 'Z577' 'Z578' 'Z579' 'Z580' 'Z581' 'Z582' 'Z583' 'Z584' 'Z585'

'Z586' 'Z588' 'Z589' 'Z590' 'Z591' 'Z592' 'Z593' 'Z594' 'Z595' 'Z596'

'Z597' 'Z598' 'Z599' 'Z600' 'Z601' 'Z602' 'Z603' 'Z604' 'Z605' 'Z608'

'Z609' 'Z610' 'Z611' 'Z612' 'Z613' 'Z614' 'Z615' 'Z616' 'Z617' 'Z618'

'Z619' 'Z620' 'Z621' 'Z622' 'Z623' 'Z624' 'Z625' 'Z626' 'Z628' 'Z629'

'Z630' 'Z631' 'Z632' 'Z633' 'Z634' 'Z635' 'Z636' 'Z638' 'Z639' 'Z640'

'Z641' 'Z642' 'Z643' 'Z644' 'Z650' 'Z651' 'Z652' 'Z653' 'Z654' 'Z655'

'Z658' 'Z659' 'Z700' 'Z701' 'Z702' 'Z703' 'Z708' 'Z709' 'Z710' 'Z711'

'Z712' 'Z713' 'Z714' 'Z715' 'Z716' 'Z717' 'Z718' 'Z719' 'Z720' 'Z721'

'Z722' 'Z723' 'Z724' 'Z725' 'Z726' 'Z728' 'Z729' 'Z730' 'Z731' 'Z732'

'Z733' 'Z734' 'Z735' 'Z736' 'Z738' 'Z739' 'Z740' 'Z741' 'Z742' 'Z743'

'Z748' 'Z749' 'Z750' 'Z755' 'Z758' 'Z759' 'Z760' 'Z761' 'Z762' 'Z763'

'Z764' 'Z765' 'Z768' 'Z769' 'Z800' 'Z801' 'Z802' 'Z803' 'Z804' 'Z805'

'Z806' 'Z807' 'Z808' 'Z809' 'Z810' 'Z811' 'Z812' 'Z813' 'Z814' 'Z818'

'Z820' 'Z821' 'Z822' 'Z823' 'Z824' 'Z825' 'Z826' 'Z827' 'Z828' 'Z830'

'Z831' 'Z832' 'Z833' 'Z834' 'Z835' 'Z836' 'Z837' 'Z840' 'Z841' 'Z842'

'Z843' 'Z848' 'Z850' 'Z851' 'Z852' 'Z853' 'Z854' 'Z855' 'Z856' 'Z857'

'Z858' 'Z859' 'Z860' 'Z862' 'Z863' 'Z865' 'Z866' 'Z867' 'Z870' 'Z872'

'Z873' 'Z874' 'Z875' 'Z876' 'Z877' 'Z878' 'Z880' 'Z881' 'Z882' 'Z883'

'Z884' 'Z885' 'Z886' 'Z887' 'Z888' 'Z889' 'Z890' 'Z891' 'Z892' 'Z893'

'Z894' 'Z895' 'Z896' 'Z897' 'Z898' 'Z899' 'Z900' 'Z901' 'Z902' 'Z903'

'Z904' 'Z905' 'Z906' 'Z907' 'Z908' 'Z910' 'Z911' 'Z912' 'Z913' 'Z914'

'Z915' 'Z916' 'Z918' 'Z920' 'Z921' 'Z923' 'Z924' 'Z925' 'Z928' 'Z929'

'Z930' 'Z931' 'Z932' 'Z933' 'Z934' 'Z935' 'Z936' 'Z938' 'Z939' 'Z940'

'Z941' 'Z942' 'Z943' 'Z944' 'Z945' 'Z946' 'Z947' 'Z948' 'Z949' 'Z950'

'Z951' 'Z952' 'Z953' 'Z954' 'Z955' 'Z958' 'Z959' 'Z960' 'Z961' 'Z962'

'Z963' 'Z964' 'Z965' 'Z967' 'Z968' 'Z969' 'Z970' 'Z971' 'Z972' 'Z973'

'Z974' 'Z975' 'Z978' 'Z980' 'Z981' 'Z982' 'Z988' 'Z990' 'Z991' 'Z992'

'Z993' 'Z998' 'Z999')) and tpf(i) eq 'C' then do;

tpf(i)='*';

CodingError = CodingError+**1**;

end;

if (substr(tdx(i),**1**,**5**)

'C4101' 'C4102' 'C7981' 'C7982' 'C7988' 'C8800' 'C8801' 'C8810' 'C8811'

'C8820' 'C8821' 'C8830' 'C8831' 'C8870' 'C8871' 'C8890' 'C8891' 'C9000'

'C9001' 'C9010' 'C9011' 'C9020' 'C9021' 'C9100' 'C9101' 'C9110' 'C9111'

'C9120' 'C9121' 'C9130' 'C9131' 'C9140' 'C9141' 'C9150' 'C9151' 'C9170'

'C9171' 'C9190' 'C9191' 'C9200' 'C9201' 'C9210' 'C9211' 'C9220' 'C9221'

'C9230' 'C9231' 'C9240' 'C9241' 'C9250' 'C9251' 'C9270' 'C9271' 'C9290'

'C9291' 'C9300' 'C9301' 'C9310' 'C9311' 'C9320' 'C9321' 'C9370' 'C9371'

'C9390' 'C9391' 'C9400' 'C9401' 'C9410' 'C9411' 'C9420' 'C9421' 'C9430'

'C9431' 'C9440' 'C9441' 'C9450' 'C9451' 'C9470' 'C9471' 'C9500' 'C9501'

'C9510' 'C9511' 'C9520' 'C9521' 'C9570' 'C9571' 'C9590' 'C9591' 'D1641'

'D1642' 'D1800' 'D1801' 'D1802' 'D1803' 'D1804' 'D1805' 'D1808' 'E0901'

'E0902' 'E1011' 'E1012' 'E1013' 'E1014' 'E1015' 'E1016' 'E1021' 'E1022'

'E1023' 'E1029' 'E1031' 'E1032' 'E1033' 'E1034' 'E1035' 'E1036' 'E1039'

'E1040' 'E1041' 'E1042' 'E1043' 'E1049' 'E1051' 'E1052' 'E1053' 'E1061'

'E1062' 'E1063' 'E1064' 'E1065' 'E1069' 'E1071' 'E1073' 'E1101' 'E1102'

'E1111' 'E1112' 'E1113' 'E1114' 'E1115' 'E1116' 'E1121' 'E1122' 'E1123'

'E1129' 'E1131' 'E1132' 'E1133' 'E1134' 'E1135' 'E1136' 'E1139' 'E1140'

'E1141' 'E1142' 'E1143' 'E1149' 'E1151' 'E1152' 'E1153' 'E1161' 'E1162'

'E1163' 'E1164' 'E1165' 'E1169' 'E1171' 'E1172' 'E1173' 'E1301' 'E1302'

'E1311' 'E1312' 'E1313' 'E1314' 'E1315' 'E1316' 'E1321' 'E1322' 'E1323'

'E1329' 'E1331' 'E1332' 'E1333' 'E1334' 'E1335' 'E1336' 'E1339' 'E1340'

'E1341' 'E1342' 'E1343' 'E1349' 'E1351' 'E1352' 'E1353' 'E1361' 'E1362'

'E1363' 'E1364' 'E1365' 'E1369' 'E1371' 'E1372' 'E1373' 'E1401' 'E1402'

'E1411' 'E1412' 'E1413' 'E1414' 'E1415' 'E1416' 'E1421' 'E1422' 'E1423'

'E1429' 'E1431' 'E1432' 'E1433' 'E1434' 'E1435' 'E1436' 'E1439' 'E1440'

'E1441' 'E1442' 'E1443' 'E1449' 'E1451' 'E1452' 'E1453' 'E1461' 'E1462'

'E1463' 'E1464' 'E1465' 'E1469' 'E1471' 'E1472' 'E1473' 'F4000' 'F4001'

'F6030' 'F6031' 'G4730' 'G4731' 'G4732' 'G4733' 'G4739' 'G5810' 'G5811'

'G5812' 'G5819' 'G8000' 'G8001' 'G8002' 'G8003' 'G8009' 'I2510' 'I2511'

'I2512' 'I2513' 'I7020' 'I7021' 'I7022' 'I7023' 'I7024' 'I9820' 'I9821'

'K3181' 'K3182' 'K4000' 'K4001' 'K4010' 'K4011' 'K4020' 'K4021' 'K4030'

'K4031' 'K4040' 'K4041' 'K4090' 'K4091' 'K5521' 'K5522' 'K5700' 'K5701'

'K5702' 'K5703' 'K5710' 'K5711' 'K5712' 'K5713' 'K5720' 'K5721' 'K5722'

'K5723' 'K5730' 'K5731' 'K5732' 'K5733' 'K5740' 'K5741' 'K5742' 'K5743'

'K5750' 'K5751' 'K5752' 'K5753' 'K5780' 'K5781' 'K5782' 'K5783' 'K5790'

'K5791' 'K5792' 'K5793' 'K6350' 'K6351' 'K6358' 'K8000' 'K8001' 'K8010'

'K8011' 'K8020' 'K8021' 'K8030' 'K8031' 'K8040' 'K8041' 'K8050' 'K8051'

'K8080' 'K8081' 'L9050' 'L9051' 'L9059' 'L9100' 'L9101' 'L9109' 'M0110'

'M0111' 'M0112' 'M0113' 'M0114' 'M0115' 'M0116' 'M0117' 'M0118' 'M0119'

'M0120' 'M0121' 'M0122' 'M0123' 'M0124' 'M0125' 'M0126' 'M0127' 'M0128'

'M0129' 'M0500' 'M0501' 'M0502' 'M0503' 'M0504' 'M0505' 'M0506' 'M0507'

'M0508' 'M0509' 'M0510' 'M0511' 'M0512' 'M0513' 'M0514' 'M0515' 'M0516'

'M0517' 'M0518' 'M0519' 'M0520' 'M0521' 'M0522' 'M0523' 'M0524' 'M0525'

'M0526' 'M0527' 'M0528' 'M0529' 'M0530' 'M0531' 'M0532' 'M0533' 'M0534'

'M0535' 'M0536' 'M0537' 'M0538' 'M0539' 'M0580' 'M0581' 'M0582' 'M0583'

'M0584' 'M0585' 'M0586' 'M0587' 'M0588' 'M0589' 'M0590' 'M0591' 'M0592'

'M0593' 'M0594' 'M0595' 'M0596' 'M0597' 'M0598' 'M0599' 'M0600' 'M0601'

'M0602' 'M0603' 'M0604' 'M0605' 'M0606' 'M0607' 'M0608' 'M0609' 'M0610'

'M0611' 'M0612' 'M0613' 'M0614' 'M0615' 'M0616' 'M0617' 'M0618' 'M0619'

'M0620' 'M0621' 'M0622' 'M0623' 'M0624' 'M0625' 'M0626' 'M0627' 'M0628' 'M0629' 'M0630' 'M0631' 'M0632' 'M0633' 'M0634' 'M0635' 'M0636' 'M0637'

'M0638' 'M0639' 'M0640' 'M0641' 'M0642' 'M0643' 'M0644' 'M0645' 'M0646'

'M0647' 'M0648' 'M0649' 'M0680' 'M0681' 'M0682' 'M0683' 'M0684' 'M0685'

'M0686' 'M0687' 'M0688' 'M0689' 'M0690' 'M0691' 'M0692' 'M0693' 'M0694'

'M0695' 'M0696' 'M0697' 'M0698' 'M0699' 'M0700' 'M0704' 'M0707' 'M0709'

'M0710' 'M0711' 'M0712' 'M0713' 'M0714' 'M0715' 'M0716' 'M0717' 'M0718'

'M0719' 'M0730' 'M0731' 'M0732' 'M0733' 'M0734' 'M0735' 'M0736' 'M0737'

'M0738' 'M0739' 'M0740' 'M0741' 'M0742' 'M0743' 'M0744' 'M0745' 'M0746'

'M0747' 'M0748' 'M0749' 'M0750' 'M0751' 'M0752' 'M0753' 'M0754' 'M0755'

'M0756' 'M0757' 'M0758' 'M0759' 'M0760' 'M0761' 'M0762' 'M0763' 'M0764'

'M0765' 'M0766' 'M0767' 'M0768' 'M0769' 'M0800' 'M0801' 'M0802' 'M0803'

'M0804' 'M0805' 'M0806' 'M0807' 'M0808' 'M0809' 'M0810' 'M0811' 'M0812'

'M0813' 'M0814' 'M0815' 'M0816' 'M0817' 'M0818' 'M0819' 'M0820' 'M0821'

'M0822' 'M0823' 'M0824' 'M0825' 'M0826' 'M0827' 'M0828' 'M0829' 'M0840'

'M0841' 'M0842' 'M0843' 'M0844' 'M0845' 'M0846' 'M0847' 'M0848' 'M0849'

'M0880' 'M0881' 'M0882' 'M0883' 'M0884' 'M0885' 'M0886' 'M0887' 'M0888'

'M0889' 'M0890' 'M0891' 'M0892' 'M0893' 'M0894' 'M0895' 'M0896' 'M0897'

'M0898' 'M0899' 'M0900' 'M0901' 'M0902' 'M0903' 'M0904' 'M0905' 'M0906'

'M0907' 'M0908' 'M0909' 'M0910' 'M0911' 'M0912' 'M0913' 'M0914' 'M0915'

'M0916' 'M0917' 'M0918' 'M0919' 'M0920' 'M0921' 'M0922' 'M0923' 'M0924'

'M0925' 'M0926' 'M0927' 'M0928' 'M0929' 'M0980' 'M0981' 'M0982' 'M0983'

'M0984' 'M0985' 'M0986' 'M0987' 'M0988' 'M0989' 'M1000' 'M1001' 'M1002'

'M1003' 'M1004' 'M1005' 'M1006' 'M1007' 'M1008' 'M1009' 'M1010' 'M1011'

'M1012' 'M1013' 'M1014' 'M1015' 'M1016' 'M1017' 'M1018' 'M1019' 'M1030'

'M1031' 'M1032' 'M1033' 'M1034' 'M1035' 'M1036' 'M1037' 'M1038' 'M1039'

'M1040' 'M1041' 'M1042' 'M1043' 'M1044' 'M1045' 'M1046' 'M1047' 'M1048'

'M1049' 'M1090' 'M1091' 'M1092' 'M1093' 'M1094' 'M1095' 'M1096' 'M1097'

'M1098' 'M1099' 'M1100' 'M1101' 'M1102' 'M1103' 'M1104' 'M1105' 'M1106'

'M1107' 'M1108' 'M1109' 'M1110' 'M1111' 'M1112' 'M1113' 'M1114' 'M1115'

'M1116' 'M1117' 'M1118' 'M1119' 'M1120' 'M1121' 'M1122' 'M1123' 'M1124'

'M1125' 'M1126' 'M1127' 'M1128' 'M1129' 'M1180' 'M1181' 'M1182' 'M1183'

'M1184' 'M1185' 'M1186' 'M1187' 'M1188' 'M1189' 'M1190' 'M1191' 'M1192'

'M1193' 'M1194' 'M1195' 'M1196' 'M1197' 'M1198' 'M1199' 'M1200' 'M1201'

'M1202' 'M1203' 'M1204' 'M1205' 'M1206' 'M1207' 'M1208' 'M1209' 'M1210'

'M1211' 'M1212' 'M1213' 'M1214' 'M1215' 'M1216' 'M1217' 'M1218' 'M1219'

'M1220' 'M1221' 'M1222' 'M1223' 'M1224' 'M1225' 'M1226' 'M1227' 'M1228'

'M1229' 'M1230' 'M1231' 'M1232' 'M1233' 'M1234' 'M1235' 'M1236' 'M1237'

'M1238' 'M1239' 'M1250' 'M1251' 'M1252' 'M1253' 'M1254' 'M1255' 'M1256'

'M1257' 'M1258' 'M1259' 'M1901' 'M1902' 'M1903' 'M1904' 'M1907' 'M1908'

'M1909' 'M1911' 'M1912' 'M1913' 'M1914' 'M1917' 'M1918' 'M1919' 'M1921'

'M1922' 'M1923' 'M1924' 'M1927' 'M1928' 'M1929' 'M1981' 'M1982' 'M1983'

'M1984' 'M1987' 'M1988' 'M1989' 'M1991' 'M1992' 'M1993' 'M1994' 'M1997'

'M1998' 'M1999' 'M2100' 'M2101' 'M2102' 'M2103' 'M2104' 'M2105' 'M2106'

'M2107' 'M2108' 'M2109' 'M2110' 'M2111' 'M2112' 'M2113' 'M2114' 'M2115'

'M2116' 'M2117' 'M2118' 'M2119' 'M2150' 'M2154' 'M2157' 'M2160' 'M2167'

'M2170' 'M2171' 'M2172' 'M2173' 'M2174' 'M2175' 'M2176' 'M2177' 'M2179'

'M2300' 'M2301' 'M2302' 'M2303' 'M2304' 'M2305' 'M2306' 'M2309' 'M2310'

'M2311' 'M2312' 'M2313' 'M2314' 'M2315' 'M2316' 'M2319' 'M2320' 'M2321'

'M2322' 'M2323' 'M2324' 'M2325' 'M2326' 'M2329' 'M2340' 'M2341' 'M2342'

'M2343' 'M2344' 'M2345' 'M2346' 'M2347' 'M2349' 'M2350' 'M2351' 'M2352'

'M2353' 'M2354' 'M2355' 'M2356' 'M2357' 'M2359' 'M2360' 'M2361' 'M2362'

'M2363' 'M2364' 'M2367' 'M2369' 'M2380' 'M2381' 'M2382' 'M2383' 'M2384'

'M2385' 'M2386' 'M2387' 'M2389' 'M2390' 'M2391' 'M2392' 'M2393' 'M2394'

'M2395' 'M2396' 'M2397' 'M2399' 'M2400' 'M2401' 'M2402' 'M2403' 'M2404'

'M2405' 'M2407' 'M2408' 'M2409' 'M2410' 'M2411' 'M2412' 'M2413' 'M2414'

'M2415' 'M2417' 'M2418' 'M2419' 'M2420' 'M2421' 'M2422' 'M2423' 'M2424'

'M2425' 'M2427' 'M2428' 'M2429' 'M2430' 'M2431' 'M2432' 'M2433' 'M2434'

'M2435' 'M2436' 'M2437' 'M2438' 'M2439' 'M2440' 'M2441' 'M2442' 'M2443'

'M2444' 'M2445' 'M2446' 'M2447' 'M2448' 'M2449' 'M2450' 'M2451' 'M2452'

'M2453' 'M2454' 'M2455' 'M2456' 'M2457' 'M2458' 'M2459' 'M2460' 'M2461'

'M2462' 'M2463' 'M2464' 'M2465' 'M2466' 'M2467' 'M2468' 'M2469' 'M2480'

'M2481' 'M2482' 'M2483' 'M2484' 'M2485' 'M2487' 'M2488' 'M2489' 'M2490'

'M2491' 'M2492' 'M2493' 'M2494' 'M2495' 'M2497' 'M2498' 'M2499' 'M2500'

'M2501' 'M2502' 'M2503' 'M2504' 'M2505' 'M2506' 'M2507' 'M2508' 'M2509'

'M2570' 'M2571' 'M2572' 'M2573' 'M2574' 'M2575' 'M2576' 'M2577' 'M2578'

'M2579' 'M4000' 'M4001' 'M4002' 'M4003' 'M4004' 'M4005' 'M4006' 'M4007'

'M4008' 'M4009' 'M4010' 'M4011' 'M4012' 'M4013' 'M4014' 'M4015' 'M4016'

'M4017' 'M4018' 'M4019' 'M4020' 'M4021' 'M4022' 'M4023' 'M4024' 'M4025'

'M4026' 'M4027' 'M4028' 'M4029' 'M4030' 'M4035' 'M4036' 'M4037' 'M4038'

'M4039' 'M4040' 'M4041' 'M4042' 'M4043' 'M4044' 'M4045' 'M4046' 'M4047'

'M4048' 'M4049' 'M4050' 'M4051' 'M4052' 'M4053' 'M4054' 'M4055' 'M4056'

'M4057' 'M4058' 'M4059' 'M4100' 'M4101' 'M4102' 'M4103' 'M4104' 'M4105'

'M4106' 'M4107' 'M4108' 'M4109' 'M4110' 'M4111' 'M4112' 'M4113' 'M4114'

'M4115' 'M4116' 'M4117' 'M4118' 'M4119' 'M4120' 'M4121' 'M4122' 'M4123'

'M4124' 'M4125' 'M4126' 'M4127' 'M4128' 'M4129' 'M4130' 'M4131' 'M4132'

'M4133' 'M4134' 'M4135' 'M4136' 'M4137' 'M4138' 'M4139' 'M4140' 'M4141'

'M4142' 'M4143' 'M4144' 'M4145' 'M4146' 'M4147' 'M4148' 'M4149' 'M4150'

'M4151' 'M4152' 'M4153' 'M4154' 'M4155' 'M4156' 'M4157' 'M4158' 'M4159'

'M4180' 'M4181' 'M4182' 'M4183' 'M4184' 'M4185' 'M4186' 'M4187' 'M4188'

'M4189' 'M4190' 'M4191' 'M4192' 'M4193' 'M4194' 'M4195' 'M4196' 'M4197'

'M4198' 'M4199' 'M4200' 'M4201' 'M4202' 'M4203' 'M4204' 'M4205' 'M4206'

'M4207' 'M4208' 'M4209' 'M4210' 'M4211' 'M4212' 'M4213' 'M4214' 'M4215'

'M4216' 'M4217' 'M4218' 'M4219' 'M4290' 'M4291' 'M4292' 'M4293' 'M4294'

'M4295' 'M4296' 'M4297' 'M4298' 'M4299' 'M4300' 'M4301' 'M4302' 'M4303'

'M4304' 'M4305' 'M4306' 'M4307' 'M4308' 'M4309' 'M4310' 'M4311' 'M4312'

'M4313' 'M4314' 'M4315' 'M4316' 'M4317' 'M4318' 'M4319' 'M4320' 'M4321'

'M4322' 'M4323' 'M4324' 'M4325' 'M4326' 'M4327' 'M4328' 'M4329' 'M4350'

'M4352' 'M4353' 'M4354' 'M4355' 'M4356' 'M4357' 'M4358' 'M4359' 'M4380'

'M4381' 'M4382' 'M4383' 'M4384' 'M4385' 'M4386' 'M4387' 'M4388' 'M4389'

'M4390' 'M4391' 'M4392' 'M4393' 'M4394' 'M4395' 'M4396' 'M4397' 'M4398'

'M4399' 'M4500' 'M4501' 'M4502' 'M4503' 'M4504' 'M4505' 'M4506' 'M4507'

'M4508' 'M4509' 'M4600' 'M4601' 'M4602' 'M4603' 'M4604' 'M4605' 'M4606'

'M4607' 'M4608' 'M4609' 'M4700' 'M4701' 'M4702' 'M4703' 'M4704' 'M4705'

'M4706' 'M4707' 'M4708' 'M4709' 'M4710' 'M4711' 'M4712' 'M4713' 'M4714'

'M4715' 'M4716' 'M4717' 'M4718' 'M4719' 'M4720' 'M4721' 'M4722' 'M4723'

'M4724' 'M4725' 'M4726' 'M4727' 'M4728' 'M4729' 'M4780' 'M4781' 'M4782'

'M4783' 'M4784' 'M4785' 'M4786' 'M4787' 'M4788' 'M4789' 'M4790' 'M4791'

'M4792' 'M4793' 'M4794' 'M4795' 'M4796' 'M4797' 'M4798' 'M4799' 'M4800'

'M4801' 'M4802' 'M4803' 'M4804' 'M4805' 'M4806' 'M4807' 'M4808' 'M4809'

'M4810' 'M4811' 'M4812' 'M4813' 'M4814' 'M4815' 'M4816' 'M4817' 'M4818'

'M4819' 'M4820' 'M4821' 'M4822' 'M4823' 'M4824' 'M4825' 'M4826' 'M4827'

'M4828' 'M4829' 'M4830' 'M4831' 'M4832' 'M4833' 'M4834' 'M4835' 'M4836'

'M4837' 'M4838' 'M4839' 'M4850' 'M4851' 'M4852' 'M4853' 'M4854' 'M4855'

'M4856' 'M4857' 'M4858' 'M4859' 'M4900' 'M4901' 'M4902' 'M4903' 'M4904'

'M4905' 'M4906' 'M4907' 'M4908' 'M4909' 'M4940' 'M4941' 'M4942' 'M4943'

'M4944' 'M4945' 'M4946' 'M4947' 'M4948' 'M4949' 'M4950' 'M4951' 'M4952'

'M4953' 'M4954' 'M4955' 'M4956' 'M4957' 'M4958' 'M4959' 'M6100' 'M6101'

'M6102' 'M6103' 'M6104' 'M6105' 'M6106' 'M6107' 'M6108' 'M6109' 'M6110'

'M6111' 'M6112' 'M6113' 'M6114' 'M6115' 'M6116' 'M6117' 'M6118' 'M6119'

'M6120' 'M6121' 'M6122' 'M6123' 'M6124' 'M6125' 'M6126' 'M6127' 'M6128'

'M6129' 'M6130' 'M6131' 'M6132' 'M6133' 'M6134' 'M6135' 'M6136' 'M6137'

'M6138' 'M6139' 'M6140' 'M6141' 'M6142' 'M6143' 'M6144' 'M6145' 'M6146'

'M6147' 'M6148' 'M6149' 'M6150' 'M6151' 'M6152' 'M6153' 'M6154' 'M6155'

'M6156' 'M6157' 'M6158' 'M6159' 'M6190' 'M6191' 'M6192' 'M6193' 'M6194'

'M6195' 'M6196' 'M6197' 'M6198' 'M6199' 'M6230' 'M6231' 'M6232' 'M6233'

'M6234' 'M6235' 'M6236' 'M6237' 'M6238' 'M6239' 'M6240' 'M6241' 'M6242'

'M6243' 'M6244' 'M6245' 'M6246' 'M6247' 'M6248' 'M6249' 'M6251' 'M6252'

'M6253' 'M6254' 'M6255' 'M6256' 'M6257' 'M6258' 'M6710' 'M6711' 'M6712'

'M6713' 'M6714' 'M6715' 'M6716' 'M6717' 'M6718' 'M6719' 'M6720' 'M6721'

'M6722' 'M6723' 'M6724' 'M6725' 'M6726' 'M6727' 'M6728' 'M6729' 'M6740'

'M6741' 'M6742' 'M6743' 'M6744' 'M6745' 'M6746' 'M6747' 'M6748' 'M6749'

'M7130' 'M7131' 'M7132' 'M7133' 'M7134' 'M7135' 'M7136' 'M7137' 'M7138'

'M7139' 'M7140' 'M7141' 'M7142' 'M7143' 'M7144' 'M7145' 'M7146' 'M7147'

'M7148' 'M7149' 'M7240' 'M7241' 'M7242' 'M7243' 'M7244' 'M7245' 'M7246'

'M7247' 'M7248' 'M7249' 'M7300' 'M7301' 'M7302' 'M7303' 'M7304' 'M7305'

'M7306' 'M7307' 'M7308' 'M7309' 'M7310' 'M7311' 'M7312' 'M7313' 'M7314'

'M7315' 'M7316' 'M7317' 'M7318' 'M7319' 'M7900' 'M7901' 'M7902' 'M7903'

'M7904' 'M7905' 'M7906' 'M7907' 'M7908' 'M7909' 'M7940' 'M7941' 'M7942'

'M7943' 'M7944' 'M7945' 'M7946' 'M7947' 'M7948' 'M7949' 'M7950' 'M7951'

'M7952' 'M7953' 'M7954' 'M7955' 'M7956' 'M7957' 'M7958' 'M7959' 'M8000'

'M8001' 'M8002' 'M8003' 'M8004' 'M8005' 'M8006' 'M8007' 'M8008' 'M8009'

'M8010' 'M8011' 'M8012' 'M8013' 'M8014' 'M8015' 'M8016' 'M8017' 'M8018'

'M8019' 'M8020' 'M8021' 'M8022' 'M8023' 'M8024' 'M8025' 'M8026' 'M8027'

'M8028' 'M8029' 'M8050' 'M8051' 'M8052' 'M8053' 'M8054' 'M8055' 'M8056'

'M8057' 'M8058' 'M8059' 'M8080' 'M8081' 'M8082' 'M8083' 'M8084' 'M8085'

'M8086' 'M8087' 'M8088' 'M8089' 'M8090' 'M8091' 'M8092' 'M8093' 'M8094'

'M8095' 'M8096' 'M8097' 'M8098' 'M8099' 'M8100' 'M8101' 'M8102' 'M8103'

'M8104' 'M8105' 'M8106' 'M8107' 'M8108' 'M8109' 'M8110' 'M8111' 'M8112'

'M8113' 'M8114' 'M8115' 'M8116' 'M8117' 'M8118' 'M8119' 'M8120' 'M8121'

'M8122' 'M8123' 'M8124' 'M8125' 'M8126' 'M8127' 'M8128' 'M8129' 'M8150'

'M8151' 'M8152' 'M8153' 'M8154' 'M8155' 'M8156' 'M8157' 'M8158' 'M8159'

'M8160' 'M8161' 'M8162' 'M8163' 'M8164' 'M8165' 'M8166' 'M8167' 'M8168'

'M8169' 'M8180' 'M8181' 'M8182' 'M8183' 'M8184' 'M8185' 'M8186' 'M8187'

'M8188' 'M8189' 'M8190' 'M8191' 'M8192' 'M8193' 'M8194' 'M8195' 'M8196'

'M8197' 'M8198' 'M8199' 'M8200' 'M8201' 'M8202' 'M8203' 'M8204' 'M8205'

'M8206' 'M8207' 'M8208' 'M8209' 'M8210' 'M8211' 'M8212' 'M8213' 'M8214'

'M8215' 'M8216' 'M8217' 'M8218' 'M8219' 'M8280' 'M8281' 'M8282' 'M8283'

'M8284' 'M8285' 'M8286' 'M8287' 'M8288' 'M8289' 'M8300' 'M8301' 'M8302'

'M8303' 'M8304' 'M8305' 'M8306' 'M8307' 'M8308' 'M8309' 'M8310' 'M8311'

'M8312' 'M8313' 'M8314' 'M8315' 'M8316' 'M8317' 'M8318' 'M8319' 'M8320'

'M8321' 'M8322' 'M8323' 'M8324' 'M8325' 'M8326' 'M8327' 'M8328' 'M8329'

'M8330' 'M8331' 'M8332' 'M8333' 'M8334' 'M8335' 'M8336' 'M8337' 'M8338'

'M8339' 'M8340' 'M8341' 'M8342' 'M8343' 'M8344' 'M8345' 'M8346' 'M8347'

'M8348' 'M8349' 'M8351' 'M8352' 'M8353' 'M8354' 'M8355' 'M8356' 'M8357'

'M8358' 'M8359' 'M8380' 'M8381' 'M8382' 'M8383' 'M8384' 'M8385' 'M8386'

'M8387' 'M8388' 'M8389' 'M8390' 'M8391' 'M8392' 'M8393' 'M8394' 'M8395'

'M8396' 'M8397' 'M8398' 'M8399' 'M8400' 'M8500' 'M8501' 'M8502' 'M8503'

'M8504' 'M8505' 'M8506' 'M8507' 'M8508' 'M8509' 'M8510' 'M8511' 'M8512'

'M8513' 'M8514' 'M8515' 'M8516' 'M8517' 'M8518' 'M8519' 'M8530' 'M8531'

'M8532' 'M8533' 'M8534' 'M8535' 'M8536' 'M8537' 'M8538' 'M8539' 'M8541'

'M8542' 'M8543' 'M8544' 'M8545' 'M8546' 'M8547' 'M8548' 'M8549' 'M8550'

'M8551' 'M8552' 'M8553' 'M8554' 'M8555' 'M8556' 'M8557' 'M8558' 'M8559'

'M8560' 'M8561' 'M8562' 'M8563' 'M8564' 'M8565' 'M8566' 'M8567' 'M8568'

'M8569' 'M8580' 'M8581' 'M8582' 'M8583' 'M8584' 'M8585' 'M8586' 'M8587'

'M8588' 'M8589' 'M8590' 'M8591' 'M8592' 'M8593' 'M8594' 'M8595' 'M8596'

'M8597' 'M8598' 'M8599' 'M8630' 'M8631' 'M8632' 'M8633' 'M8634' 'M8635'

'M8636' 'M8637' 'M8638' 'M8639' 'M8640' 'M8641' 'M8642' 'M8643' 'M8644'

'M8645' 'M8646' 'M8647' 'M8648' 'M8649' 'M8650' 'M8651' 'M8652' 'M8653'

'M8654' 'M8655' 'M8656' 'M8657' 'M8658' 'M8659' 'M8660' 'M8661' 'M8662'

'M8663' 'M8664' 'M8665' 'M8666' 'M8667' 'M8668' 'M8669' 'M8700' 'M8701'

'M8702' 'M8703' 'M8704' 'M8705' 'M8706' 'M8707' 'M8708' 'M8709' 'M8710'

'M8711' 'M8712' 'M8713' 'M8714' 'M8715' 'M8716' 'M8717' 'M8718' 'M8719'

'M8720' 'M8721' 'M8722' 'M8723' 'M8724' 'M8725' 'M8726' 'M8727' 'M8728'

'M8729' 'M8730' 'M8731' 'M8732' 'M8733' 'M8734' 'M8735' 'M8736' 'M8737'

'M8738' 'M8739' 'M8780' 'M8781' 'M8782' 'M8783' 'M8784' 'M8785' 'M8786'

'M8787' 'M8788' 'M8789' 'M8790' 'M8791' 'M8792' 'M8793' 'M8794' 'M8795'

'M8796' 'M8797' 'M8798' 'M8799' 'M8880' 'M8881' 'M8882' 'M8883' 'M8884'

'M8885' 'M8886' 'M8887' 'M8888' 'M8900' 'M8901' 'M8902' 'M8903' 'M8904'

'M8905' 'M8906' 'M8907' 'M8908' 'M8909' 'M8910' 'M8911' 'M8912' 'M8913'

'M8914' 'M8915' 'M8916' 'M8917' 'M8918' 'M8919' 'M8920' 'M8921' 'M8922'

'M8923' 'M8924' 'M8925' 'M8926' 'M8927' 'M8928' 'M8929' 'M8930' 'M8931'

'M8932' 'M8933' 'M8934' 'M8935' 'M8936' 'M8937' 'M8938' 'M8939' 'M8940'

'M8941' 'M8942' 'M8943' 'M8944' 'M8945' 'M8946' 'M8947' 'M8948' 'M8949'

'M8950' 'M8951' 'M8952' 'M8953' 'M8954' 'M8955' 'M8956' 'M8957' 'M8958'

'M8959' 'M8960' 'M8961' 'M8962' 'M8963' 'M8964' 'M8965' 'M8966' 'M8967'

'M8968' 'M8969' 'M9000' 'M9001' 'M9002' 'M9003' 'M9004' 'M9005' 'M9006'

'M9007' 'M9008' 'M9009' 'M9010' 'M9011' 'M9012' 'M9013' 'M9014' 'M9015'

'M9016' 'M9017' 'M9018' 'M9019' 'M9020' 'M9021' 'M9022' 'M9023' 'M9024'

'M9025' 'M9026' 'M9027' 'M9028' 'M9029' 'M9030' 'M9031' 'M9032' 'M9033'

'M9034' 'M9035' 'M9036' 'M9037' 'M9038' 'M9039' 'M9040' 'M9041' 'M9042'

'M9043' 'M9044' 'M9045' 'M9046' 'M9047' 'M9048' 'M9049' 'M9050' 'M9051'

'M9052' 'M9053' 'M9054' 'M9055' 'M9056' 'M9057' 'M9058' 'M9059' 'M9060'

'M9061' 'M9062' 'M9063' 'M9064' 'M9065' 'M9066' 'M9067' 'M9068' 'M9069'

'M9080' 'M9081' 'M9082' 'M9083' 'M9084' 'M9085' 'M9086' 'M9087' 'M9088'

'M9089' 'M9320' 'M9321' 'M9322' 'M9323' 'M9324' 'M9325' 'M9326' 'M9327'

'M9329' 'M9420' 'M9421' 'M9422' 'M9423' 'M9424' 'M9425' 'M9426' 'M9427'

'M9428' 'M9429' 'M9430' 'M9431' 'M9432' 'M9433' 'M9434' 'M9435' 'M9436'

'M9437' 'M9438' 'M9439' 'M9480' 'M9481' 'M9482' 'M9483' 'M9484' 'M9485'

'M9486' 'M9487' 'M9488' 'M9489' 'M9490' 'M9491' 'M9492' 'M9493' 'M9494'

'M9495' 'M9496' 'M9497' 'M9498' 'M9499' 'N1890' 'N1891' 'O2411' 'O2412'

'O2419' 'O2421' 'O2422' 'O2429' 'O2431' 'O2432' 'O2439' 'O2441' 'O2442'

'O2449' 'O2451' 'O2452' 'O2459' 'O2491' 'O2492' 'O2499' 'P0701' 'P0702'

'P0703' 'P0711' 'P0712' 'P0713' 'P0721' 'P0722' 'P0730' 'P0731' 'P0732'

'Q0000' 'Q0001' 'Q0009' 'Q0020' 'Q0021' 'Q0022' 'Q0181' 'Q0182' 'Q0183'

'Q0184' 'Q0189' 'Q0400' 'Q0401' 'Q0409' 'Q0431' 'Q0432' 'Q0433' 'Q0434'

'Q0435' 'Q0436' 'Q0439' 'Q0460' 'Q0461' 'Q0462' 'Q0500' 'Q0501' 'Q0502'

'Q0510' 'Q0511' 'Q0512' 'Q0520' 'Q0521' 'Q0522' 'Q0530' 'Q0531' 'Q0532'

'Q0540' 'Q0541' 'Q0542' 'Q0550' 'Q0551' 'Q0552' 'Q0560' 'Q0561' 'Q0562'

'Q0570' 'Q0571' 'Q0572' 'Q0580' 'Q0581' 'Q0582' 'Q0590' 'Q0591' 'Q0592'

'Q0781' 'Q0782' 'Q0789' 'Q2100' 'Q2101' 'Q2102' 'Q2109' 'Q2110' 'Q2111'

'Q2112' 'Q2119' 'Q2241' 'Q2242' 'Q2301' 'Q2302' 'Q2321' 'Q2322' 'Q3911'

'Q3912' 'Q3919' 'Q3921' 'Q3922' 'Q3981' 'Q3982' 'Q3989' 'Q4200' 'Q4201'

'Q4202' 'Q4203' 'Q4204' 'Q4205' 'Q4209' 'Q4220' 'Q4221' 'Q4222' 'Q4229'

'Q4310' 'Q4311' 'Q4312' 'Q4319' 'Q4331' 'Q4332' 'Q4339' 'Q4471' 'Q4479'

'Q4531' 'Q4539' 'Q4581' 'Q4582' 'Q4583' 'Q4584' 'Q4589' 'Q5000' 'Q5001'

'Q5002' 'Q5010' 'Q5011' 'Q5012' 'Q5031' 'Q5039' 'Q5061' 'Q5069' 'Q5300'

'Q5301' 'Q5302' 'Q5303' 'Q5309' 'Q5310' 'Q5311' 'Q5312' 'Q5313' 'Q5319'

'Q5320' 'Q5321' 'Q5322' 'Q5323' 'Q5329' 'Q5390' 'Q5391' 'Q5392' 'Q5393'

'Q5399' 'Q5500' 'Q5501' 'Q5502' 'Q5521' 'Q5522' 'Q5529' 'Q6140' 'Q6141'

'Q6142' 'Q6150' 'Q6151' 'Q6152' 'Q6211' 'Q6212' 'Q6213' 'Q6214' 'Q6218'

'Q6219' 'Q6231' 'Q6232' 'Q6233' 'Q6234' 'Q6239' 'Q6251' 'Q6252' 'Q6259'

'Q6260' 'Q6261' 'Q6262' 'Q6263' 'Q6264' 'Q6265' 'Q6266' 'Q6269' 'Q6270'

'Q6271' 'Q6272' 'Q6301' 'Q6302' 'Q6309' 'Q6310' 'Q6311' 'Q6319' 'Q6320'

'Q6321' 'Q6322' 'Q6323' 'Q6329' 'Q6381' 'Q6389' 'Q6411' 'Q6419' 'Q6420'

'Q6421' 'Q6422' 'Q6431' 'Q6432' 'Q6433' 'Q6434' 'Q6439' 'Q6441' 'Q6442'

'Q6443' 'Q6449' 'Q6471' 'Q6472' 'Q6473' 'Q6474' 'Q6475' 'Q6476' 'Q6477'

'Q6478' 'Q6479' 'Q6560' 'Q6561' 'Q6562' 'Q6681' 'Q6689' 'Q6741' 'Q6742'

'Q6749' 'Q6751' 'Q6752' 'Q6759' 'Q6921' 'Q6929' 'Q7131' 'Q7132' 'Q7133'

'Q7231' 'Q7232' 'Q7233' 'Q7380' 'Q7389' 'Q7401' 'Q7402' 'Q7403' 'Q7404'

'Q7405' 'Q7406' 'Q7407' 'Q7408' 'Q7409' 'Q7481' 'Q7482' 'Q7483' 'Q7484'

'Q7485' 'Q7489' 'Q7501' 'Q7502' 'Q7503' 'Q7504' 'Q7505' 'Q7506' 'Q7509'

'Q7531' 'Q7539' 'Q7581' 'Q7589' 'Q7621' 'Q7622' 'Q7631' 'Q7639' 'Q7641'

'Q7642' 'Q7643' 'Q7644' 'Q7645' 'Q7649' 'Q7661' 'Q7662' 'Q7663' 'Q7669'

'Q7671' 'Q7672' 'Q7679' 'Q7700' 'Q7701' 'Q7702' 'Q7703' 'Q7709' 'Q7781'

'Q7782' 'Q7789' 'Q8481' 'Q8489' 'Q8581' 'Q8582' 'Q8583' 'Q8584' 'Q8589'

'Q8681' 'Q8682' 'Q8683' 'Q8684' 'Q8685' 'Q8686' 'Q8687' 'Q8689' 'Q8701'

'Q8702' 'Q8703' 'Q8704' 'Q8705' 'Q8706' 'Q8707' 'Q8709' 'Q8711' 'Q8712'

'Q8713' 'Q8714' 'Q8715' 'Q8716' 'Q8717' 'Q8718' 'Q8719' 'Q8721' 'Q8722'

'Q8723' 'Q8724' 'Q8725' 'Q8726' 'Q8727' 'Q8729' 'Q8731' 'Q8732' 'Q8733'

'Q8739' 'Q8781' 'Q8782' 'Q8783' 'Q8784' 'Q8785' 'Q8789' 'Q8901' 'Q8909'

'Q8911' 'Q8912' 'Q8919' 'Q8921' 'Q8922' 'Q8923' 'Q8924' 'Q8925' 'Q8926'

'Q8929' 'Q8930' 'Q8931' 'Q8932' 'Q8933' 'Q8934' 'Q8935' 'Q8939' 'Q8941'

'Q8942' 'Q8943' 'Q8944' 'Q8945' 'Q8946' 'Q8949' 'Q8971' 'Q8979' 'Q8981'

'Q8989' 'U5000' 'U5001' 'U5002' 'U5003' 'U5004' 'U5005' 'U5008' 'U5009'

'U5030' 'U5039' 'U5120' 'U5121' 'U5122' 'U5123' 'U5128' 'U5129' 'U5400'

'U5401' 'U5402' 'U5408' 'U5409' 'U5410' 'U5418' 'U5419' 'U5450' 'U5451'

'U5459' 'U5520' 'U5521' 'U5522' 'U5523' 'U5528' 'U5529' 'U5600' 'U5601'

'U5630' 'U5631' 'U5632' 'U5633' 'U5634' 'U5635' 'U5636' 'U5637' 'U5638'

'U5639' 'U5700' 'U5701' 'U5702' 'U5703' 'U5704' 'U5705' 'U5706' 'U5707'

'U5708' 'U5709' 'U6020' 'U6021' 'U6022' 'U6029' 'U6130' 'U6131' 'U6132'

'U6133' 'U6134' 'U6135' 'U6136' 'U6138' 'U6139' 'U6140' 'U6141' 'U6142'

'U6148' 'U6149' 'U6301' 'U6302' 'U6303' 'U6308' 'U6309' 'U6600' 'U6601'

'U6602' 'U6603' 'U6608' 'U6609' 'U6640' 'U6649' 'U6740' 'U6741' 'U6742'

'U6749' 'U7300' 'U7301' 'U7302' 'U7303' 'U7304' 'U7305' 'U7306' 'U7307'

'U7308' 'U7309' 'V2000' 'V2001' 'V2002' 'V2008' 'V2009' 'V2010' 'V2011'

'V2012' 'V2018' 'V2019' 'V2020' 'V2021' 'V2022' 'V2028' 'V2029' 'V2030'

'V2031' 'V2032' 'V2038' 'V2039' 'V2040' 'V2041' 'V2042' 'V2048' 'V2049'

'V2050' 'V2051' 'V2052' 'V2058' 'V2059' 'V2090' 'V2091' 'V2092' 'V2098'

'V2099' 'V2100' 'V2101' 'V2102' 'V2108' 'V2109' 'V2110' 'V2111' 'V2112'

'V2118' 'V2119' 'V2120' 'V2121' 'V2122' 'V2128' 'V2129' 'V2130' 'V2131'

'V2132' 'V2138' 'V2139' 'V2140' 'V2141' 'V2142' 'V2148' 'V2149' 'V2150'

'V2151' 'V2152' 'V2158' 'V2159' 'V2190' 'V2191' 'V2192' 'V2198' 'V2199'

'V2200' 'V2201' 'V2202' 'V2208' 'V2209' 'V2210' 'V2211' 'V2212' 'V2218'

'V2219' 'V2220' 'V2221' 'V2222' 'V2228' 'V2229' 'V2230' 'V2231' 'V2232'

'V2238' 'V2239' 'V2240' 'V2241' 'V2242' 'V2248' 'V2249' 'V2250' 'V2251'

'V2252' 'V2258' 'V2259' 'V2290' 'V2291' 'V2292' 'V2298' 'V2299' 'V2300'

'V2301' 'V2302' 'V2308' 'V2309' 'V2310' 'V2311' 'V2312' 'V2318' 'V2319'

'V2320' 'V2321' 'V2322' 'V2328' 'V2329' 'V2330' 'V2331' 'V2332' 'V2338'

'V2339' 'V2340' 'V2341' 'V2342' 'V2348' 'V2349' 'V2350' 'V2351' 'V2352'

'V2358' 'V2359' 'V2390' 'V2391' 'V2392' 'V2398' 'V2399' 'V2400' 'V2401'

'V2402' 'V2408' 'V2409' 'V2410' 'V2411' 'V2412' 'V2418' 'V2419' 'V2420'

'V2421' 'V2422' 'V2428' 'V2429' 'V2430' 'V2431' 'V2432' 'V2438' 'V2439'

'V2440' 'V2441' 'V2442' 'V2448' 'V2449' 'V2450' 'V2451' 'V2452' 'V2458'

'V2459' 'V2490' 'V2491' 'V2492' 'V2498' 'V2499' 'V2500' 'V2501' 'V2502'

'V2508' 'V2509' 'V2510' 'V2511' 'V2512' 'V2518' 'V2519' 'V2520' 'V2521'

'V2522' 'V2528' 'V2529' 'V2530' 'V2531' 'V2532' 'V2538' 'V2539' 'V2540'

'V2541' 'V2542' 'V2548' 'V2549' 'V2550' 'V2551' 'V2552' 'V2558' 'V2559'

'V2590' 'V2591' 'V2592' 'V2598' 'V2599' 'V2600' 'V2601' 'V2602' 'V2608'

'V2609' 'V2610' 'V2611' 'V2612' 'V2618' 'V2619' 'V2620' 'V2621' 'V2622'

'V2628' 'V2629' 'V2630' 'V2631' 'V2632' 'V2638' 'V2639' 'V2640' 'V2641'

'V2642' 'V2648' 'V2649' 'V2650' 'V2651' 'V2652' 'V2658' 'V2659' 'V2690'

'V2691' 'V2692' 'V2698' 'V2699' 'V2700' 'V2701' 'V2702' 'V2708' 'V2709'

'V2710' 'V2711' 'V2712' 'V2718' 'V2719' 'V2720' 'V2721' 'V2722' 'V2728'

'V2729' 'V2730' 'V2731' 'V2732' 'V2738' 'V2739' 'V2740' 'V2741' 'V2742'

'V2748' 'V2749' 'V2750' 'V2751' 'V2752' 'V2758' 'V2759' 'V2790' 'V2791'

'V2792' 'V2798' 'V2799' 'V2800' 'V2801' 'V2802' 'V2808' 'V2809' 'V2810'

'V2811' 'V2812' 'V2818' 'V2819' 'V2820' 'V2821' 'V2822' 'V2828' 'V2829'

'V2830' 'V2831' 'V2832' 'V2838' 'V2839' 'V2840' 'V2841' 'V2842' 'V2848'

'V2849' 'V2850' 'V2851' 'V2852' 'V2858' 'V2859' 'V2890' 'V2891' 'V2892'

'V2898' 'V2899' 'V8000' 'V8001' 'V8009' 'V8600' 'V8601' 'V8602' 'V8609'

'V8610' 'V8611' 'V8612' 'V8619' 'V8620' 'V8621' 'V8622' 'V8629' 'V8630'

'V8631' 'V8632' 'V8639' 'V8640' 'V8641' 'V8642' 'V8649' 'V8650' 'V8651'

'V8652' 'V8659' 'V8660' 'V8661' 'V8662' 'V8669' 'V8670' 'V8671' 'V8672'

'V8679' 'V8690' 'V8691' 'V8692' 'V8699' 'W3020' 'W3021' 'W3022' 'W3023'

'W3024' 'W3028' 'W3029' 'X2000' 'X2001' 'X2002' 'X2003' 'X2004' 'X2005'

'X2008' 'X2009' 'X2600' 'X2601' 'X2602' 'X2608' 'X2609' 'X2610' 'X2618'

'X9210' 'X9211' 'X9212' 'X9214' 'X9216' 'X9217' 'X9218' 'X9219' 'X9220'

'X9221' 'X9222' 'X9224' 'X9226' 'X9227' 'X9228' 'X9229' 'Y0300' 'Y0301'

'Y0302' 'Y0303' 'Y0304' 'Y0305' 'Y0306' 'Y0307' 'Y0308' 'Y0309' 'Y0310'

'Y0311' 'Y0312' 'Y0313' 'Y0314' 'Y0315' 'Y0316' 'Y0317' 'Y0318' 'Y0319'

'Y0320' 'Y0321' 'Y0322' 'Y0323' 'Y0324' 'Y0325' 'Y0326' 'Y0327' 'Y0328'

'Y0329' 'Y0330' 'Y0331' 'Y0332' 'Y0333' 'Y0334' 'Y0335' 'Y0336' 'Y0337'

'Y0338' 'Y0339' 'Y0340' 'Y0341' 'Y0342' 'Y0343' 'Y0344' 'Y0345' 'Y0346'

'Y0347' 'Y0348' 'Y0349' 'Y0350' 'Y0351' 'Y0352' 'Y0353' 'Y0354' 'Y0355'

'Y0356' 'Y0357' 'Y0358' 'Y0359' 'Y0380' 'Y0381' 'Y0382' 'Y0383' 'Y0384'

'Y0385' 'Y0386' 'Y0387' 'Y0388' 'Y0389' 'Y0390' 'Y0391' 'Y0392' 'Y0393'

'Y0394' 'Y0395' 'Y0396' 'Y0397' 'Y0398' 'Y0399' 'Y3501' 'Y3502' 'Y3503'

'Y3504' 'Y3505' 'Y3509' 'Y3641' 'Y3642' 'Y3643' 'Y3644' 'Y3645' 'Y3649'

'Y9200' 'Y9209' 'Y9210' 'Y9211' 'Y9212' 'Y9213' 'Y9218' 'Y9219' 'Y9221'

'Y9230' 'Y9231' 'Y9232' 'Y9233' 'Y9234' 'Y9235' 'Y9236' 'Y9238' 'Y9239'

'Y9240' 'Y9241' 'Y9242' 'Y9248' 'Y9249' 'Y9250' 'Y9251' 'Y9252' 'Y9253'

'Y9258' 'Y9259' 'Y9260' 'Y9261' 'Y9262' 'Y9263' 'Y9264' 'Y9265' 'Y9266'

'Y9268' 'Y9269' 'Y9280' 'Y9281' 'Y9282' 'Y9283' 'Y9284' 'Y9285' 'Y9286'

'Y9287' 'Y9288' 'Z1381' 'Z1382' 'Z1383' 'Z1384' 'Z1385' 'Z1386' 'Z1388'

'Z2251' 'Z2252' 'Z2259' 'Z3551' 'Z3552' 'Z3900' 'Z3901' 'Z3902' 'Z3903'

'Z4000' 'Z4001' 'Z4008' 'Z5160' 'Z5161' 'Z5162' 'Z5163' 'Z5164' 'Z5169'

'Z5181' 'Z5188' 'Z5200' 'Z5208' 'Z6370' 'Z6371' 'Z6372' 'Z6379' 'Z8610'

'Z8611' 'Z8612' 'Z8613' 'Z8618' 'Z8641' 'Z8642' 'Z8643' 'Z8710' 'Z8711'

'Z8712' 'Z8718' 'Z9221' 'Z9222' 'Z9228' 'Z9660' 'Z9661' 'Z9662' 'Z9663'

'Z9664' 'Z9665' 'Z9666' 'Z9668'))

and tpf(i) eq 'C' then

do;

tpf(i)='*';

CodingError = CodingError+**1**;

end;

if (tdx(i) in

‘80000' 'M80001' 'M80002' 'M80003' 'M80006' 'M80009' 'M80010' 'M80011'

'M80013' 'M80021' 'M80022' 'M80023' 'M80026' 'M80029' 'M80031' 'M80032'

'M80033' 'M80036' 'M80039' 'M80040' 'M80041' 'M80042' 'M80043' 'M80046'

'M80049' 'M80050' 'M80051' 'M80052' 'M80053' 'M80056' 'M80059' 'M80100'

'M80101' 'M80102' 'M80103' 'M80106' 'M80109' 'M80110' 'M80111' 'M80112'

'M80113' 'M80116' 'M80119' 'M80122' 'M80123' 'M80126' 'M80129' 'M80132'

'M80133' 'M80136' 'M80139' 'M80142' 'M80143' 'M80146' 'M80149' 'M80152'

'M80153' 'M80156' 'M80159' 'M80202' 'M80203' 'M80206' 'M80209' 'M80212'

'M80213' 'M80216' 'M80219' 'M80222' 'M80223' 'M80226' 'M80229' 'M80302'

'M80303' 'M80306' 'M80309' 'M80312' 'M80313' 'M80316' 'M80319' 'M80322'

'M80323' 'M80326' 'M80329' 'M80331' 'M80332' 'M80333' 'M80336' 'M80339'

'M80341' 'M80342' 'M80343' 'M80346' 'M80349' 'M80352' 'M80353' 'M80356'

'M80359' 'M80400' 'M80401' 'M80412' 'M80413' 'M80416' 'M80419' 'M80423'

'M80426' 'M80429' 'M80433' 'M80436' 'M80439' 'M80443' 'M80446' 'M80449'

'M80453' 'M80456' 'M80459' 'M80463' 'M80466' 'M80469' 'M80500' 'M80501'

'M80502' 'M80503' 'M80506' 'M80509' 'M80510' 'M80511' 'M80513' 'M80516'

'M80519' 'M80520' 'M80521' 'M80522' 'M80523' 'M80526' 'M80529' 'M80530'

'M80531' 'M80600' 'M80601' 'M80701' 'M80702' 'M80703' 'M80706' 'M80709'

'M80712' 'M80713' 'M80716' 'M80719' 'M80721' 'M80722' 'M80723' 'M80726'

'M80729' 'M80731' 'M80732' 'M80733' 'M80736' 'M80739' 'M80741' 'M80742'

'M80743' 'M80746' 'M80749' 'M80751' 'M80752' 'M80753' 'M80756' 'M80759'

'M80762' 'M80763' 'M80772' 'M80782' 'M80783' 'M80786' 'M80789' 'M80802'

'M80812' 'M80823' 'M80826' 'M80829' 'M80831' 'M80832' 'M80833' 'M80836'

'M80839' 'M80841' 'M80842' 'M80843' 'M80846' 'M80849' 'M80901' 'M80902'

'M80903' 'M80906' 'M80909' 'M80912' 'M80913' 'M80916' 'M80919' 'M80922'

'M80923' 'M80926' 'M80929' 'M80931' 'M80932' 'M80933' 'M80936' 'M80939'

'M80941' 'M80942' 'M80943' 'M80946' 'M80949' 'M80952' 'M80953' 'M80956'

'M80959' 'M80960' 'M80971' 'M80972' 'M80973' 'M80976' 'M80979' 'M80981'

'M80982' 'M80983' 'M80986' 'M80989' 'M81000' 'M81001' 'M81003' 'M81006'

'M81009' 'M81010' 'M81011' 'M81013' 'M81016' 'M81019' 'M81020' 'M81021'

'M81023' 'M81026' 'M81029' 'M81030' 'M81031' 'M81033' 'M81036' 'M81039'

'M81100' 'M81101' 'M81103' 'M81106' 'M81109' 'M81200' 'M81201' 'M81202'

'M81203' 'M81206' 'M81209' 'M81210' 'M81211' 'M81213' 'M81216' 'M81219'

'M81221' 'M81223' 'M81226' 'M81229' 'M81232' 'M81233' 'M81236' 'M81239'

'M81242' 'M81243' 'M81246' 'M81249' 'M81300' 'M81301' 'M81302' 'M81303'

'M81306' 'M81309' 'M81312' 'M81313' 'M81316' 'M81319' 'M81400' 'M81401'

'M81402' 'M81403' 'M81406' 'M81409' 'M81413' 'M81416' 'M81419' 'M81423'

'M81426' 'M81429' 'M81432' 'M81433' 'M81436' 'M81439' 'M81442' 'M81443'

'M81446' 'M81449' 'M81452' 'M81453' 'M81456' 'M81459' 'M81460' 'M81470'

'M81473' 'M81476' 'M81479' 'M81482' 'M81490' 'M81500' 'M81501' 'M81503'

'M81506' 'M81509' 'M81510' 'M81511' 'M81513' 'M81516' 'M81519' 'M81521'

'M81523' 'M81526' 'M81529' 'M81531' 'M81533' 'M81536' 'M81539' 'M81541'

'M81543' 'M81546' 'M81549' 'M81551' 'M81553' 'M81556' 'M81559' 'M81561'

'M81563' 'M81566' 'M81569' 'M81571' 'M81573' 'M81576' 'M81579' 'M81600'

'M81601' 'M81602' 'M81603' 'M81606' 'M81609' 'M81610' 'M81611' 'M81612'

'M81613' 'M81616' 'M81619' 'M81623' 'M81626' 'M81629' 'M81700' 'M81701'

'M81703' 'M81706' 'M81709' 'M81713' 'M81716' 'M81719' 'M81723' 'M81726'

'M81729' 'M81733' 'M81736' 'M81739' 'M81743' 'M81746' 'M81749' 'M81753'

'M81756' 'M81759' 'M81802' 'M81803' 'M81806' 'M81809' 'M81900' 'M81902'

'M81903' 'M81906' 'M81909' 'M81910' 'M81911' 'M82000' 'M82001' 'M82002'

'M82003' 'M82006' 'M82009' 'M82012' 'M82013' 'M82016' 'M82019' 'M82020'

'M82040' 'M82100' 'M82101' 'M82102' 'M82103' 'M82106' 'M82109' 'M82110'

'M82111' 'M82113' 'M82116' 'M82119' 'M82120' 'M82121' 'M82130' 'M82131'

'M82141' 'M82142' 'M82143' 'M82146' 'M82149' 'M82152' 'M82153' 'M82156'

'M82159' 'M82200' 'M82202' 'M82203' 'M82206' 'M82209' 'M82210' 'M82211'

'M82212' 'M82213' 'M82216' 'M82219' 'M82302' 'M82303' 'M82306' 'M82309'

'M82313' 'M82316' 'M82319' 'M82401' 'M82403' 'M82406' 'M82409' 'M82411'

'M82413' 'M82416' 'M82419' 'M82421' 'M82423' 'M82426' 'M82429' 'M82431'

'M82433' 'M82436' 'M82439' 'M82441' 'M82443' 'M82446' 'M82449' 'M82451'

'M82453' 'M82456' 'M82459' 'M82463' 'M82466' 'M82469' 'M82473' 'M82476'

'M82479' 'M82481' 'M82493' 'M82496' 'M82499' 'M82501' 'M82503' 'M82506'

'M82509' 'M82510' 'M82513' 'M82516' 'M82519' 'M82521' 'M82522' 'M82523'

'M82526' 'M82529' 'M82531' 'M82532' 'M82533' 'M82536' 'M82539' 'M82541'

'M82542' 'M82543' 'M82546' 'M82549' 'M82551' 'M82552' 'M82553' 'M82556'

'M82559' 'M82600' 'M82601' 'M82602' 'M82603' 'M82606' 'M82609' 'M82610'

'M82611' 'M82612' 'M82613' 'M82616' 'M82619' 'M82622' 'M82623' 'M82626'

'M82629' 'M82630' 'M82631' 'M82632' 'M82633' 'M82636' 'M82639' 'M82640'

'M82700' 'M82701' 'M82703' 'M82706' 'M82709' 'M82710' 'M82713' 'M82716'

'M82720' 'M82721' 'M82723' 'M82726' 'M82729' 'M82800' 'M82801' 'M82803'

'M82806' 'M82809' 'M82810' 'M82811' 'M82813' 'M82816' 'M82819' 'M82900'

'M82901' 'M82903' 'M82906' 'M82909' 'M83000' 'M83001' 'M83003' 'M83006'

'M83009' 'M83100' 'M83101' 'M83103' 'M83106' 'M83109' 'M83111' 'M83121'

'M83122' 'M83123' 'M83126' 'M83129' 'M83130' 'M83131' 'M83133' 'M83136'

'M83139' 'M83143' 'M83146' 'M83149' 'M83153' 'M83156' 'M83159' 'M83161'

'M83163' 'M83166' 'M83169' 'M83171' 'M83173' 'M83176' 'M83179' 'M83181'

'M83183' 'M83186' 'M83189' 'M83191' 'M83193' 'M83196' 'M83199' 'M83203'

'M83206' 'M83209' 'M83210' 'M83211' 'M83213' 'M83216' 'M83219' 'M83220'

'M83221' 'M83223' 'M83226' 'M83229' 'M83230' 'M83231' 'M83232' 'M83233'

'M83236' 'M83239' 'M83240' 'M83241' 'M83250' 'M83251' 'M83300' 'M83301'

'M83302' 'M83303' 'M83306' 'M83309' 'M83312' 'M83313' 'M83316' 'M83319'

'M83322' 'M83323' 'M83326' 'M83329' 'M83330' 'M83331' 'M83332' 'M83333'

'M83336' 'M83339' 'M83340' 'M83341' 'M83353' 'M83356' 'M83359' 'M83360'

'M83361' 'M83371' 'M83372' 'M83373' 'M83376' 'M83379' 'M83401' 'M83402'

'M83403' 'M83406' 'M83409' 'M83411' 'M83412' 'M83413' 'M83416' 'M83419'

'M83421' 'M83422' 'M83423' 'M83426' 'M83429' 'M83431' 'M83432' 'M83433'

'M83436' 'M83439' 'M83441' 'M83442' 'M83443' 'M83446' 'M83449' 'M83451'

'M83452' 'M83453' 'M83456' 'M83459' 'M83461' 'M83462' 'M83463' 'M83466'

'M83469' 'M83471' 'M83472' 'M83473' 'M83476' 'M83479' 'M83501' 'M83502'

'M83503' 'M83506' 'M83509' 'M83601' 'M83610' 'M83613' 'M83616' 'M83619'

'M83700' 'M83701' 'M83702' 'M83703' 'M83706' 'M83709' 'M83710' 'M83711'

'M83720' 'M83721' 'M83730' 'M83731' 'M83740' 'M83741' 'M83750' 'M83751'

'M83800' 'M83801' 'M83802' 'M83803' 'M83806' 'M83809' 'M83810' 'M83811'

'M83813' 'M83816' 'M83819' 'M83823' 'M83833' 'M83836' 'M83839' 'M83843'

'M83846' 'M83849' 'M83900' 'M83901' 'M83903' 'M83906' 'M83909' 'M83910'

'M83911' 'M83920' 'M83921' 'M84000' 'M84001' 'M84002' 'M84003' 'M84006'

'M84009' 'M84010' 'M84011' 'M84012' 'M84013' 'M84016' 'M84019' 'M84020'

'M84021' 'M84023' 'M84026' 'M84029' 'M84030' 'M84031' 'M84032' 'M84033'

'M84036' 'M84039' 'M84040' 'M84041' 'M84050' 'M84051' 'M84060' 'M84061'

'M84070' 'M84071' 'M84073' 'M84076' 'M84079' 'M84080' 'M84081' 'M84083'

'M84086' 'M84089' 'M84090' 'M84091' 'M84093' 'M84096' 'M84099' 'M84100'

'M84101' 'M84102' 'M84103' 'M84106' 'M84109' 'M84132' 'M84133' 'M84136'

'M84139' 'M84200' 'M84201' 'M84202' 'M84203' 'M84206' 'M84209' 'M84301'

'M84302' 'M84303' 'M84306' 'M84309' 'M84400' 'M84401' 'M84402' 'M84403'

'M84406' 'M84409' 'M84410' 'M84411' 'M84412' 'M84413' 'M84416' 'M84419'

'M84421' 'M84430' 'M84441' 'M84500' 'M84502' 'M84503' 'M84506' 'M84509'

'M84511' 'M84521' 'M84523' 'M84526' 'M84529' 'M84530' 'M84531' 'M84532'

'M84533' 'M84536' 'M84539' 'M84540' 'M84600' 'M84601' 'M84602' 'M84603'

'M84606' 'M84609' 'M84610' 'M84612' 'M84613' 'M84616' 'M84619' 'M84621'

'M84631' 'M84700' 'M84701' 'M84702' 'M84703' 'M84706' 'M84709' 'M84710'

'M84712' 'M84713' 'M84716' 'M84719' 'M84721' 'M84731' 'M84800' 'M84801'

'M84803' 'M84806' 'M84809' 'M84813' 'M84816' 'M84819' 'M84823' 'M84826'

'M84829' 'M84903' 'M84906' 'M84909' 'M85002' 'M85003' 'M85006' 'M85009'

'M85012' 'M85013' 'M85016' 'M85019' 'M85022' 'M85023' 'M85026' 'M85029'

'M85030' 'M85032' 'M85033' 'M85036' 'M85039' 'M85040' 'M85041' 'M85042'

'M85043' 'M85046' 'M85049' 'M85050' 'M85051' 'M85060' 'M85061' 'M85072'

'M85082' 'M85083' 'M85086' 'M85089' 'M85102' 'M85103' 'M85106' 'M85109'

'M85122' 'M85123' 'M85126' 'M85129' 'M85132' 'M85133' 'M85136' 'M85139'

'M85142' 'M85143' 'M85146' 'M85149' 'M85202' 'M85203' 'M85206' 'M85209'

'M85212' 'M85213' 'M85216' 'M85219' 'M85222' 'M85223' 'M85226' 'M85229'

'M85233' 'M85236' 'M85239' 'M85243' 'M85246' 'M85249' 'M85252' 'M85253'

'M85256' 'M85259' 'M85303' 'M85306' 'M85309' 'M85402' 'M85403' 'M85406'

'M85409' 'M85412' 'M85413' 'M85416' 'M85419' 'M85422' 'M85423' 'M85426'

'M85429' 'M85432' 'M85433' 'M85436' 'M85439' 'M85500' 'M85501' 'M85503'

'M85506' 'M85509' 'M85513' 'M85516' 'M85519' 'M85600' 'M85602' 'M85603'

'M85606' 'M85609' 'M85610' 'M85611' 'M85623' 'M85626' 'M85629' 'M85702'

'M85703' 'M85706' 'M85709' 'M85712' 'M85713' 'M85716' 'M85719' 'M85722'

'M85723' 'M85726' 'M85729' 'M85732' 'M85733' 'M85736' 'M85739' 'M85742'

'M85743' 'M85746' 'M85749' 'M85752' 'M85753' 'M85756' 'M85759' 'M85762'

'M85763' 'M85766' 'M85769' 'M85800' 'M85801' 'M85803' 'M85806' 'M85809'

'M85811' 'M85813' 'M85816' 'M85819' 'M85821' 'M85823' 'M85826' 'M85829'

'M85831' 'M85833' 'M85836' 'M85839' 'M85841' 'M85843' 'M85846' 'M85849'

'M85851' 'M85853' 'M85856' 'M85859' 'M85862' 'M85863' 'M85866' 'M85869'

'M85870' 'M85882' 'M85883' 'M85886' 'M85889' 'M85893' 'M85896' 'M85899'

'M85901' 'M85903' 'M85906' 'M85909' 'M85911' 'M85913' 'M85916' 'M85919'

'M85921' 'M85923' 'M85926' 'M85929' 'M85931' 'M85933' 'M85936' 'M85939'

'M86000' 'M86001' 'M86003' 'M86006' 'M86009' 'M86010' 'M86020' 'M86100'

'M86101' 'M86103' 'M86106' 'M86109' 'M86201' 'M86203' 'M86206' 'M86209'

'M86211' 'M86221' 'M86231' 'M86300' 'M86301' 'M86303' 'M86306' 'M86309'

'M86310' 'M86311' 'M86313' 'M86316' 'M86319' 'M86321' 'M86323' 'M86326'

'M86329' 'M86331' 'M86341' 'M86343' 'M86346' 'M86349' 'M86401' 'M86403'

'M86406' 'M86409' 'M86410' 'M86421' 'M86500' 'M86501' 'M86503' 'M86506'

'M86509' 'M86600' 'M86700' 'M86703' 'M86706' 'M86709' 'M86710' 'M86711'

'M86800' 'M86801' 'M86803' 'M86806' 'M86809' 'M86811' 'M86821' 'M86830'

'M86901' 'M86903' 'M86906' 'M86909' 'M86911' 'M86913' 'M86916' 'M86919'

'M86921' 'M86923' 'M86926' 'M86929' 'M86931' 'M86933' 'M86936' 'M86939'

'M87000' 'M87003' 'M87006' 'M87009' 'M87103' 'M87106' 'M87109' 'M87110'

'M87111' 'M87113' 'M87116' 'M87119' 'M87120' 'M87123' 'M87126' 'M87129'

'M87130' 'M87200' 'M87201' 'M87202' 'M87203' 'M87206' 'M87209' 'M87213'

'M87216' 'M87219' 'M87220' 'M87221' 'M87222' 'M87223' 'M87226' 'M87229'

'M87230' 'M87232' 'M87233' 'M87236' 'M87239' 'M87250' 'M87251' 'M87260'

'M87261' 'M87270' 'M87272' 'M87273' 'M87276' 'M87279' 'M87280' 'M87281'

'M87282' 'M87283' 'M87286' 'M87289' 'M87300' 'M87301' 'M87302' 'M87303'

'M87306' 'M87309' 'M87400' 'M87401' 'M87402' 'M87403' 'M87406' 'M87409'

'M87411' 'M87412' 'M87413' 'M87416' 'M87419' 'M87421' 'M87422' 'M87423'

'M87426' 'M87429' 'M87432' 'M87433' 'M87436' 'M87439' 'M87442' 'M87443'

'M87446' 'M87449' 'M87452' 'M87453' 'M87456' 'M87459' 'M87462' 'M87463'

'M87466' 'M87469' 'M87500' 'M87501' 'M87600' 'M87601' 'M87610' 'M87611'

'M87612' 'M87613' 'M87616' 'M87619' 'M87621' 'M87700' 'M87702' 'M87703'

'M87706' 'M87709' 'M87710' 'M87711' 'M87712' 'M87713' 'M87716' 'M87719'

'M87720' 'M87721' 'M87722' 'M87723' 'M87726' 'M87729' 'M87732' 'M87733'

'M87736' 'M87739' 'M87742' 'M87743' 'M87746' 'M87749' 'M87800' 'M87801'

'M87803' 'M87806' 'M87809' 'M87900' 'M87901' 'M88000' 'M88003' 'M88006'

'M88009' 'M88011' 'M88013' 'M88016' 'M88019' 'M88021' 'M88023' 'M88026'

'M88029' 'M88033' 'M88036' 'M88039' 'M88043' 'M88046' 'M88049' 'M88053'

'M88056' 'M88059' 'M88063' 'M88066' 'M88069' 'M88100' 'M88101' 'M88103'

'M88106' 'M88109' 'M88110' 'M88111' 'M88113' 'M88116' 'M88119' 'M88120'

'M88121' 'M88123' 'M88126' 'M88129' 'M88130' 'M88131' 'M88133' 'M88136'

'M88139' 'M88141' 'M88143' 'M88146' 'M88149' 'M88150' 'M88151' 'M88153'

'M88156' 'M88159' 'M88200' 'M88211' 'M88213' 'M88216' 'M88219' 'M88221'

'M88223' 'M88226' 'M88229' 'M88230' 'M88231' 'M88240' 'M88241' 'M88250'

'M88251' 'M88253' 'M88256' 'M88259' 'M88260' 'M88261' 'M88271' 'M88273'

'M88276' 'M88279' 'M88300' 'M88301' 'M88303' 'M88306' 'M88309' 'M88310'

'M88311' 'M88320' 'M88321' 'M88323' 'M88326' 'M88329' 'M88333' 'M88341'

'M88351' 'M88353' 'M88356' 'M88359' 'M88361' 'M88363' 'M88366' 'M88369'

'M88400' 'M88403' 'M88406' 'M88409' 'M88411' 'M88413' 'M88416' 'M88419'

'M88420' 'M88421' 'M88500' 'M88501' 'M88503' 'M88506' 'M88509' 'M88510'

'M88511' 'M88513' 'M88516' 'M88519' 'M88520' 'M88521' 'M88523' 'M88526'

'M88529' 'M88533' 'M88536' 'M88539' 'M88540' 'M88543' 'M88546' 'M88549'

'M88553' 'M88556' 'M88559' 'M88560' 'M88570' 'M88571' 'M88573' 'M88576'

'M88579' 'M88583' 'M88600' 'M88601' 'M88603' 'M88606' 'M88609' 'M88610'

'M88611' 'M88700' 'M88701' 'M88800' 'M88801' 'M88803' 'M88806' 'M88809'

'M88810' 'M88900' 'M88901' 'M88903' 'M88906' 'M88909' 'M88910' 'M88911'

'M88913' 'M88916' 'M88919' 'M88920' 'M88921' 'M88930' 'M88931' 'M88940'

'M88941' 'M88943' 'M88946' 'M88949' 'M88950' 'M88951' 'M88953' 'M88956'

'M88959' 'M88963' 'M88966' 'M88969' 'M88971' 'M88981' 'M89000' 'M89001'

'M89003' 'M89006' 'M89009' 'M89013' 'M89016' 'M89019' 'M89023' 'M89026'

'M89029' 'M89030' 'M89031' 'M89040' 'M89041' 'M89050' 'M89051' 'M89103'

'M89106' 'M89109' 'M89123' 'M89126' 'M89129' 'M89203' 'M89206' 'M89209'

'M89213' 'M89216' 'M89219' 'M89300' 'M89301' 'M89303' 'M89306' 'M89309'

'M89311' 'M89313' 'M89316' 'M89319' 'M89320' 'M89333' 'M89336' 'M89339'

'M89343' 'M89346' 'M89349' 'M89350' 'M89351' 'M89353' 'M89356' 'M89359'

'M89360' 'M89361' 'M89363' 'M89366' 'M89369' 'M89400' 'M89401' 'M89403'

'M89406' 'M89409' 'M89413' 'M89416' 'M89419' 'M89501' 'M89503' 'M89506'

'M89509' 'M89511' 'M89513' 'M89516' 'M89519' 'M89590' 'M89591' 'M89593'

'M89596' 'M89599' 'M89601' 'M89603' 'M89606' 'M89609' 'M89633' 'M89636'

'M89639' 'M89643' 'M89646' 'M89649' 'M89650' 'M89651' 'M89660' 'M89661'

'M89670' 'M89671' 'M89673' 'M89676' 'M89679' 'M89703' 'M89706' 'M89709'

'M89713' 'M89716' 'M89719' 'M89723' 'M89726' 'M89729' 'M89733' 'M89736'

'M89739' 'M89741' 'M89743' 'M89746' 'M89749' 'M89803' 'M89806' 'M89809'

'M89813' 'M89816' 'M89819' 'M89820' 'M89821' 'M89823' 'M89826' 'M89829'

'M89830' 'M89831' 'M89900' 'M89901' 'M89903' 'M89906' 'M89909' 'M89913'

'M89916' 'M89919' 'M90000' 'M90001' 'M90003' 'M90006' 'M90009' 'M90100'

'M90101' 'M90103' 'M90106' 'M90109' 'M90110' 'M90111' 'M90120' 'M90121'

'M90130' 'M90131' 'M90140' 'M90141' 'M90143' 'M90146' 'M90149' 'M90150'

'M90151' 'M90153' 'M90156' 'M90159' 'M90160' 'M90200' 'M90201' 'M90203'

'M90206' 'M90209' 'M90300' 'M90301' 'M90400' 'M90401' 'M90403' 'M90406'

'M90409' 'M90413' 'M90416' 'M90419' 'M90423' 'M90426' 'M90429' 'M90433'

'M90436' 'M90439' 'M90443' 'M90446' 'M90449' 'M90500' 'M90501' 'M90503'

'M90506' 'M90509' 'M90510' 'M90511' 'M90513' 'M90516' 'M90519' 'M90520'

'M90521' 'M90523' 'M90526' 'M90529' 'M90533' 'M90536' 'M90539' 'M90540'

'M90541' 'M90550' 'M90551' 'M90553' 'M90556' 'M90559' 'M90603' 'M90606'

'M90609' 'M90612' 'M90613' 'M90616' 'M90619' 'M90623' 'M90626' 'M90629'

'M90632' 'M90633' 'M90636' 'M90639' 'M90642' 'M90643' 'M90646' 'M90649'

'M90652' 'M90653' 'M90656' 'M90659' 'M90702' 'M90703' 'M90706' 'M90709'

'M90713' 'M90716' 'M90719' 'M90723' 'M90726' 'M90729' 'M90731' 'M90733'

'M90736' 'M90739' 'M90800' 'M90801' 'M90803' 'M90806' 'M90809' 'M90813'

'M90816' 'M90819' 'M90823' 'M90826' 'M90829' 'M90833' 'M90836' 'M90839'

'M90840' 'M90841' 'M90843' 'M90846' 'M90849' 'M90851' 'M90853' 'M90856'

'M90859' 'M90900' 'M90901' 'M90903' 'M90906' 'M90909' 'M90911' 'M90913'

'M90916' 'M90919' 'M91000' 'M91001' 'M91003' 'M91006' 'M91009' 'M91013'

'M91016' 'M91019' 'M91023' 'M91026' 'M91029' 'M91030' 'M91041' 'M91043'

'M91046' 'M91049' 'M91053' 'M91056' 'M91059' 'M91100' 'M91101' 'M91103'

'M91106' 'M91109' 'M91200' 'M91201' 'M91203' 'M91206' 'M91209' 'M91210'

'M91220' 'M91230' 'M91243' 'M91246' 'M91249' 'M91250' 'M91300' 'M91301'

'M91303' 'M91306' 'M91309' 'M91310' 'M91311' 'M91320' 'M91321' 'M91323'

'M91326' 'M91329' 'M91331' 'M91333' 'M91336' 'M91339' 'M91351' 'M91353'

'M91356' 'M91359' 'M91361' 'M91363' 'M91366' 'M91369' 'M91403' 'M91406'

'M91409' 'M91410' 'M91420' 'M91500' 'M91501' 'M91503' 'M91506' 'M91509'

'M91600' 'M91601' 'M91610' 'M91611' 'M91613' 'M91616' 'M91619' 'M91700'

'M91701' 'M91703' 'M91706' 'M91709' 'M91710' 'M91711' 'M91720' 'M91721'

'M91730' 'M91731' 'M91740' 'M91741' 'M91750' 'M91751' 'M91753' 'M91756'

'M91759' 'M91800' 'M91801' 'M91803' 'M91806' 'M91809' 'M91813' 'M91816'

'M91819' 'M91823' 'M91826' 'M91829' 'M91833' 'M91836' 'M91839' 'M91843'

'M91846' 'M91849' 'M91853' 'M91856' 'M91859' 'M91863' 'M91866' 'M91869'

'M91873' 'M91876' 'M91879' 'M91910' 'M91911' 'M91923' 'M91926' 'M91929'

'M91933' 'M91936' 'M91939' 'M91943' 'M91946' 'M91949' 'M91953' 'M91956'

'M91959' 'M92000' 'M92001' 'M92100' 'M92101' 'M92200' 'M92201' 'M92203'

'M92206' 'M92209' 'M92210' 'M92211' 'M92213' 'M92216' 'M92219' 'M92300'

'M92301' 'M92303' 'M92306' 'M92309' 'M92313' 'M92316' 'M92319' 'M92403'

'M92406' 'M92409' 'M92410' 'M92411' 'M92423' 'M92426' 'M92429' 'M92433'

'M92436' 'M92439' 'M92500' 'M92501' 'M92503' 'M92506' 'M92509' 'M92510'

'M92511' 'M92513' 'M92516' 'M92519' 'M92520' 'M92521' 'M92523' 'M92526'

'M92529' 'M92603' 'M92606' 'M92609' 'M92613' 'M92616' 'M92619' 'M92620'

'M92621' 'M92700' 'M92701' 'M92703' 'M92706' 'M92709' 'M92710' 'M92711'

'M92720' 'M92721' 'M92730' 'M92740' 'M92741' 'M92750' 'M92800' 'M92801'

'M92810' 'M92820' 'M92821' 'M92900' 'M92901' 'M92903' 'M92906' 'M92909'

'M93000' 'M93001' 'M93010' 'M93011' 'M93020' 'M93100' 'M93101' 'M93103'

'M93106' 'M93109' 'M93110' 'M93111' 'M93120' 'M93121' 'M93200' 'M93201'

'M93210' 'M93211' 'M93220' 'M93300' 'M93301' 'M93303' 'M93306' 'M93309'

'M93400' 'M93401' 'M93411' 'M93423' 'M93426' 'M93429' 'M93501' 'M93511'

'M93521' 'M93600' 'M93601' 'M93610' 'M93611' 'M93621' 'M93623' 'M93626'

'M93629' 'M93630' 'M93631' 'M93643' 'M93646' 'M93649' 'M93653' 'M93656'

'M93659' 'M93700' 'M93701' 'M93703' 'M93706' 'M93709' 'M93710' 'M93711'

'M93713' 'M93716' 'M93719' 'M93721' 'M93723' 'M93726' 'M93729' 'M93730'

'M93731' 'M93803' 'M93806' 'M93809' 'M93813' 'M93816' 'M93819' 'M93821'

'M93823' 'M93826' 'M93829' 'M93831' 'M93841' 'M93900' 'M93901' 'M93903'

'M93906' 'M93909' 'M93910' 'M93911' 'M93913' 'M93916' 'M93919' 'M93923'

'M93926' 'M93929' 'M93933' 'M93936' 'M93939' 'M93941' 'M93943' 'M93946'

'M93949' 'M94003' 'M94006' 'M94009' 'M94013' 'M94016' 'M94019' 'M94103'

'M94106' 'M94109' 'M94113' 'M94116' 'M94119' 'M94121' 'M94130' 'M94203'

'M94211' 'M94213' 'M94216' 'M94219' 'M94233' 'M94236' 'M94239' 'M94243'

'M94246' 'M94249' 'M94303' 'M94306' 'M94309' 'M94401' 'M94403' 'M94406'

'M94409' 'M94413' 'M94416' 'M94419' 'M94421' 'M94423' 'M94426' 'M94429'

'M94441' 'M94443' 'M94446' 'M94449' 'M94501' 'M94503' 'M94506' 'M94509'

'M94513' 'M94516' 'M94519' 'M94603' 'M94606' 'M94609' 'M94703' 'M94706'

'M94709' 'M94713' 'M94716' 'M94719' 'M94723' 'M94726' 'M94729' 'M94733'

'M94736' 'M94739' 'M94743' 'M94746' 'M94749' 'M94803' 'M94806' 'M94809'

'M94900' 'M94901' 'M94903' 'M94906' 'M94909' 'M94910' 'M94911' 'M94920'

'M94921' 'M94930' 'M94931' 'M95001' 'M95003' 'M95006' 'M95009' 'M95010'

'M95013' 'M95016' 'M95019' 'M95020' 'M95023' 'M95026' 'M95029' 'M95030'

'M95031' 'M95033' 'M95036' 'M95039' 'M95043' 'M95046' 'M95049' 'M95051'

'M95053' 'M95056' 'M95059' 'M95061' 'M95070' 'M95073' 'M95076' 'M95079'

'M95083' 'M95086' 'M95089' 'M95100' 'M95103' 'M95106' 'M95109' 'M95113'

'M95116' 'M95119' 'M95123' 'M95126' 'M95129' 'M95133' 'M95136' 'M95139'

'M95141' 'M95201' 'M95203' 'M95206' 'M95209' 'M95211' 'M95213' 'M95216'

'M95219' 'M95223' 'M95226' 'M95229' 'M95230' 'M95231' 'M95233' 'M95236'

'M95239' 'M95300' 'M95301' 'M95303' 'M95306' 'M95309' 'M95310' 'M95311'

'M95313' 'M95316' 'M95319' 'M95320' 'M95321' 'M95330' 'M95331' 'M95333'

'M95336' 'M95339' 'M95340' 'M95341' 'M95343' 'M95346' 'M95349' 'M95350'

'M95351' 'M95370' 'M95371' 'M95380' 'M95381' 'M95383' 'M95386' 'M95389'

'M95391' 'M95393' 'M95396' 'M95399' 'M95400' 'M95401' 'M95403' 'M95406'

'M95409' 'M95410' 'M95411' 'M95500' 'M95501' 'M95503' 'M95506' 'M95509'

'M95600' 'M95601' 'M95603' 'M95606' 'M95609' 'M95613' 'M95616' 'M95619'

'M95620' 'M95700' 'M95701' 'M95710' 'M95711' 'M95713' 'M95716' 'M95719'

'M95800' 'M95801' 'M95803' 'M95806' 'M95809' 'M95813' 'M95816' 'M95819'

'M95820' 'M95821' 'M95903' 'M95913' 'M95963' 'M96503' 'M96513' 'M96523'

'M96533' 'M96543' 'M96553' 'M96593' 'M96613' 'M96623' 'M96633' 'M96643'

'M96653' 'M96673' 'M96703' 'M96713' 'M96733' 'M96753' 'M96783' 'M96793'

'M96803' 'M96843' 'M96873' 'M96893' 'M96903' 'M96913' 'M96953' 'M96983'

'M96993' 'M97003' 'M97013' 'M97023' 'M97053' 'M97083' 'M97093' 'M97143'

'M97163' 'M97173' 'M97183' 'M97193' 'M97273' 'M97283' 'M97293' 'M97313'

'M97323' 'M97333' 'M97343' 'M97401' 'M97403' 'M97413' 'M97423' 'M97503'

'M97511' 'M97521' 'M97531' 'M97543' 'M97553' 'M97563' 'M97573' 'M97583'

'M97603' 'M97613' 'M97623' 'M97643' 'M97651' 'M97661' 'M97671' 'M97681'

'M97691' 'M98003' 'M98013' 'M98053' 'M98203' 'M98233' 'M98263' 'M98273'

'M98313' 'M98323' 'M98333' 'M98343' 'M98353' 'M98363' 'M98373' 'M98403'

'M98603' 'M98613' 'M98633' 'M98663' 'M98673' 'M98703' 'M98713' 'M98723'

'M98733' 'M98743' 'M98753' 'M98763' 'M98913' 'M98953' 'M98963' 'M98973'

'M99103' 'M99203' 'M99303' 'M99313' 'M99403' 'M99453' 'M99463' 'M99483'

'M99503' 'M99603' 'M99613' 'M99623' 'M99633' 'M99643' 'M99701' 'M99751'

'M99803' 'M99823' 'M99833' 'M99843' 'M99853' 'M99863' 'M99873' 'M99893'))

and tpf(i) eq 'C' then

do;

tpf(i)='*';

CodingError = CodingError+**1**;

end;

end;

**run**;

**proc** **summary** data = Combine nway missing;

class CodingError

;

var sepn;

output out = S1 (drop = _type_ _freq_) sum=;

**run**;
