# Supplementary material for: Development of a validation algorithm for 'present on admission' flagging
Source: BMC Med Inform Decis Mak. 2009 Dec 1;9:48. doi: 10.1186/1472-6947-9-48 (PMC2793244; doi:10.1186/1472-6947-9-48)
Supplement: Additional file 2 — Instructions for data cleaning algorithm 20090304. This document includes additional information for use of the data cleaning algorithm. [file 1472-6947-9-48-S2.DOC]

**Data cleaning algorithm: description and instructions**

This routine has been designed for use with SAS data files which contain one record for each patient discharge that includes left justified ICD-10-AM diagnosis (TDIAG) codes and a ‘condition present on admission’ flag (TPREF) for each diagnosis code. The data cleaning algorithm identifies diagnosis codes unlikely to arise after admission that have been flagged as not present on admission (‘C’).[[1]](#footnote-2) The algorithm substitutes the ‘C’ flag with an asterisk.

**Code features:**

The routine creates an array of all the diagnosis code fields together. The Victorian Admitted Episodes Dataset (VAED) has a total of 40 diagnosis fields labelled tdiag1, tdiag2, tdiag3……up to tdiag40.

array tdx(**40**) tdiag1-tdiag40;

A matching array is created for the condition present on admission fields together. The VAED has a total of 40 condition present on admission (CPOA) fields labelled tpref1, tpref2, tpref3……up to tpref40 each corresponding to the 40 diagnosis fields.

array tpf(**40**) tpref1-tpref40;

Other datasets will have different combinations of diagnosis code variables and labels. [[2]](#footnote-3)

The diagnosis code identification is divided into four parts which reflect the different code lengths; 3 character, 4 character, 5 character and 6 character codes.

if (substr(tdx(i),**1**,**3**) in

The above starts at the first position in the diagnosis code and searches for a 3 character string (1,3).

Once a diagnosis code has been identified the routine tests the corresponding CPOA field for conditions arising after admission (‘C’ flag).

and tpf(i) eq 'C'

If both of these conditions are met the CPOA ‘C’ flag is replaced with an asterisk (‘*’)..

then

do;

tpref(i)='*';

The resultant SAS dataset (‘Combine’) is a variant of the original dataset(s) where invalid CPOA flags are replaced with an ‘*’.

**Estimating the number of coding errors**

As well as replacing the invalid flags the routine counts the number of times a condition not present on admission flag is replaced.

CodingError = CodingError+1;

The number of replacements for each discharge record is summarised into a table (S1)

**proc** **summary** data = Combine nway missing;

class CodingError;

.var sepn;

output out = S1 (drop = _type_ _freq_) sum=;

run;

The resultant report lists the number of coding errors and the frequency of discharges with that number of errors. For example in the table below 2,094,528 discharges had zero flagging errors and 2,652 discharges each contained 2 flagging errors:

| Coding Error | sepn |
| --- | --- |
| 0 | 2094528 |
| 1 | 19858 |
| 2 | 2652 |

**Steps to follow for use of the algorithm with other datasets:**

1. As our code was run on the Victorian Admitted Episodes Dataset (VAED) we entered the file name ‘H:\Vic data\vaed_06_07’. Update with the relevant file name and path.
2. Change ‘tdiag’ to name of the fields that refer to diagnosis codes; change numbering to reflect the maximum number of diagnoses recorded.
3. Update ‘tpref’ to the name of the field which stores the CPOA flags; change numbering to reflect the maximum number of CPOA flags recorded, as for diagnoses.
4. Update the ‘C’ to reflect the character which indicates a condition ‘not present on admission’.
5. If you do not want the code to replace invalid codes with an ‘*’ change the asterisk to a number/character of your choice.
6. Run the code in SAS.

1. We have kept this routine relatively linear to help with comprehension. [↑](#footnote-ref-2)
2. For example a dataset with a principal diagnosis (pdiag_1) and 24 other diagnoses (odiag_1, odiag_2, … odiag_24) would be created with the following code:

   array tdx(**40**) pdiag_1 odiag_1-odiag_24; [↑](#footnote-ref-3)
